# Deletion of the diabetes candidate gene *Slc16a13* in mice attenuates diet-induced ectopic lipid accumulation and insulin resistance

**DOI:** 10.1038/s42003-021-02279-8

**Published:** 2021-07-01

**Authors:** Tina Schumann, Jörg König, Christian von Loeffelholz, Daniel F. Vatner, Dongyan Zhang, Rachel J. Perry, Michel Bernier, Jason Chami, Christine Henke, Anica Kurzbach, Nermeen N. El-Agroudy, Diana M. Willmes, Dominik Pesta, Rafael de Cabo, John F. O´Sullivan, Eric Simon, Gerald I. Shulman, Bradford S. Hamilton, Andreas L. Birkenfeld

**Affiliations:** 1 Section of Metabolic and Vascular Medicine, Medical Clinic III, Dresden University School of Medicine, Technische Universität Dresden, Dresden, Germany; 2 Paul Langerhans Institute Dresden of the Helmholtz Center Munich at University Hospital and Faculty of Medicine, Technische Universität Dresden, Dresden, Germany; 3 German Center for Diabetes Research (DZD), Neuherberg, Germany; 4 Clinical Pharmacology and Clinical Toxicology, Institute of Experimental and Clinical Pharmacology and Toxicology, Friedrich-Alexander-Universität Erlangen-Nürnberg, Erlangen, Germany; 5 Department of Anaesthesiology and Intensive Care, Jena University Hospital, Jena, Germany; 6 Department of Internal Medicine, Yale School of Medicine, New Haven, CT USA; 7 Department of Cellular and Molecular Physiology, Yale School of Medicine, New Haven, CT USA; 8 Experimental Gerontology Section, Translational Gerontology Branch, National Institute on Aging, National Institutes of Health, Baltimore, MD USA; 9 Heart Research Institute, Newtown, NSW Australia; 10 Institute of Aerospace Medicine, German Aerospace Center (DLR), Cologne, Germany; 11 Centre for Endocrinology, Diabetes and Preventive Medicine (CEDP), University Hospital Cologne, Cologne, Germany; 12 Institute for Clinical Diabetology, German Diabetes Center, Leibniz Center for Diabetes Research at Heinrich-Heine University Düsseldorf, Düsseldorf, Germany; 13 Charles Perkins Centre, The University of Sydney, Camperdown, NSW Australia; 14 Department of Cardiology, Royal Prince Alfred Hospital, Camperdown, NSW Australia; 15 Computational Biology, Boehringer-Ingelheim Pharma GmbH & Co. KG, Biberach an der Riss, Germany; 16 CardioMetabolic Diseases Research, Boehringer-Ingelheim Pharma GmbH & Co. KG, Biberach an der Riss, Germany; 17 King’s College London, Department of Diabetes, School of Life Course Science, London, UK; 18 Institute for Diabetes Research and Metabolic Diseases of the Helmholtz Centre Munich at the University of Tübingen, Tübingen, Germany; 19 Department of Endocrinology, Diabetology and Nephrology, University Hospital of Tübingen, Tübingen, Germany

**Keywords:** Type 2 diabetes, Fat metabolism, Animal disease models, Non-alcoholic fatty liver disease

## Abstract

Genome-wide association studies have identified *SLC16A13* as a novel susceptibility gene for type 2 diabetes. The *SLC16A13* gene encodes SLC16A13/MCT13, a member of the solute carrier 16 family of monocarboxylate transporters. Despite its potential importance to diabetes development, the physiological function of SLC16A13 is unknown. Here, we validate Slc16a13 as a lactate transporter expressed at the plasma membrane and report on the effect of *Slc16a13* deletion in a mouse model. We show that loss of Slc16a13 increases mitochondrial respiration in the liver, leading to reduced hepatic lipid accumulation and increased hepatic insulin sensitivity in high-fat diet fed *Slc16a13* knockout mice. We propose a mechanism for improved hepatic insulin sensitivity in the context of Slc16a13 deficiency in which reduced intrahepatocellular lactate availability drives increased AMPK activation and increased mitochondrial respiration, while reducing hepatic lipid content. Slc16a13 deficiency thereby attenuates hepatic diacylglycerol-PKCε mediated insulin resistance in obese mice. Together, these data suggest that SLC16A13 is a potential target for the treatment of type 2 diabetes and non-alcoholic fatty liver disease.

## Abstract (web summary)

Schumann et al. demonstrate that the loss of a lactate transporter Slc16a13 increases mitochondrial respiration in the liver, which reduces hepatic lipid accumulation while increasing hepatic insulin sensitivity in mice fed a high-fat diet. This study suggests SLC16A13 as a potential target for the treatment of type 2 diabetes and non-alcoholic fatty liver disease.

## Introduction

Obesity is the major risk factor for type 2 diabetes (T2D) and is associated with ectopic fat accumulation in the liver (non-alcoholic fatty liver disease, NAFLD) and insulin resistance. T2D is an expanding international epidemic. Even with modern therapies, diabetes-associated mortality is 2–3 times higher as compared with non-affected individuals^1^. Therefore, more efficient therapies to target the root cause of NAFLD are an urgent clinical need, and research on novel therapeutic targets is of high interest. More than 140 gene loci have been linked to T2D, including members of the solute carrier (SLC) superfamily^2^. In 2014, a novel locus was identified in people of Mexican origin that associates with T2D at the genome-wide significance and spans the two adjacent genes *SLC16A11* and *SLC16A13*, encoding for solute carriers SLC16A11/MCT11 and SLC16A13/MCT13^3^. The SLC16 family of monocarboxylate transporters (MCT) mediates fluxes of monocarboxylates, e.g., l-lactate, pyruvate, and ketone bodies, across the plasma membrane and is crucial for cellular energy metabolism^4,5^. While SLC16A11 is known to be a pyruvate transporter^6^, SLC16A13 is one of the orphan members of the SLC16 family. The strongest association signal in Mexicans with T2D was ultimately localized to the *SLC16A11* gene, establishing it as a gene of great interest to the broader scientific community. Subsequent genome-wide association studies (GWAS) recently validated the *SLC16A11* risk haplotype^7–12^ and most research focused on providing evidence for a causal relationship between the development of diabetes and changes in SLC16A11 function. Yet, in mouse models, this relationship is currently a subject of controversy. While AAV-mediated *Slc16a11* knockdown in high-fat diet (HFD)-fed wild-type mice leads to improvement in glucose tolerance and hepatic insulin signaling and lowered hepatic lipid accumulation^13^, AAV-mediated reconstitution of mutated *Slc16a11* in the liver of knockout mice worsens the organismal response to high-fat diet^14^. Especially disagreements whether T2D-associated *SLC16A11* variants reflect loss- or gain-of-function mutations arose^15,16^. Therefore, it seems reasonable to also investigate SLC16A13 in the context of T2D susceptibility; besides, the function and role of SLC16A13 in metabolic disease are completely unknown. Of note, *SLC16A13* was linked to T2D in another GWAS, describing the polymorphism *rs312457* associated with T2D in people of Japanese origin^17^. Upregulation of murine *Slc16a13* in the small intestine after treatment with an agonist of PPARα^18^, a key regulator of hepatic lipid metabolism, supports the idea that SLC16A13 may also be involved in energy homeostasis. Since the human phenotype associated with the *SLC16A13* polymorphisms reflects higher susceptibility to T2D, we hypothesized that changes in SLC16A13 abundance will affect hepatic lipid deposition and insulin sensitivity. Our objective was to determine the physiological function of the SLC16A13 transporter and to elucidate how transporter deficiency affects the development of metabolic dysfunction. Therefore, we studied *SLC16A13* expression in human and mouse tissue and generated gain- and loss-of-function models, including *Slc16a13* knockout mice that were metabolically characterized in the context of diet-induced obesity.

## Results

### Hepatic SLC16A13 is upregulated under conditions of metabolic dysfunction in humans and mice

**Fig. 1:**
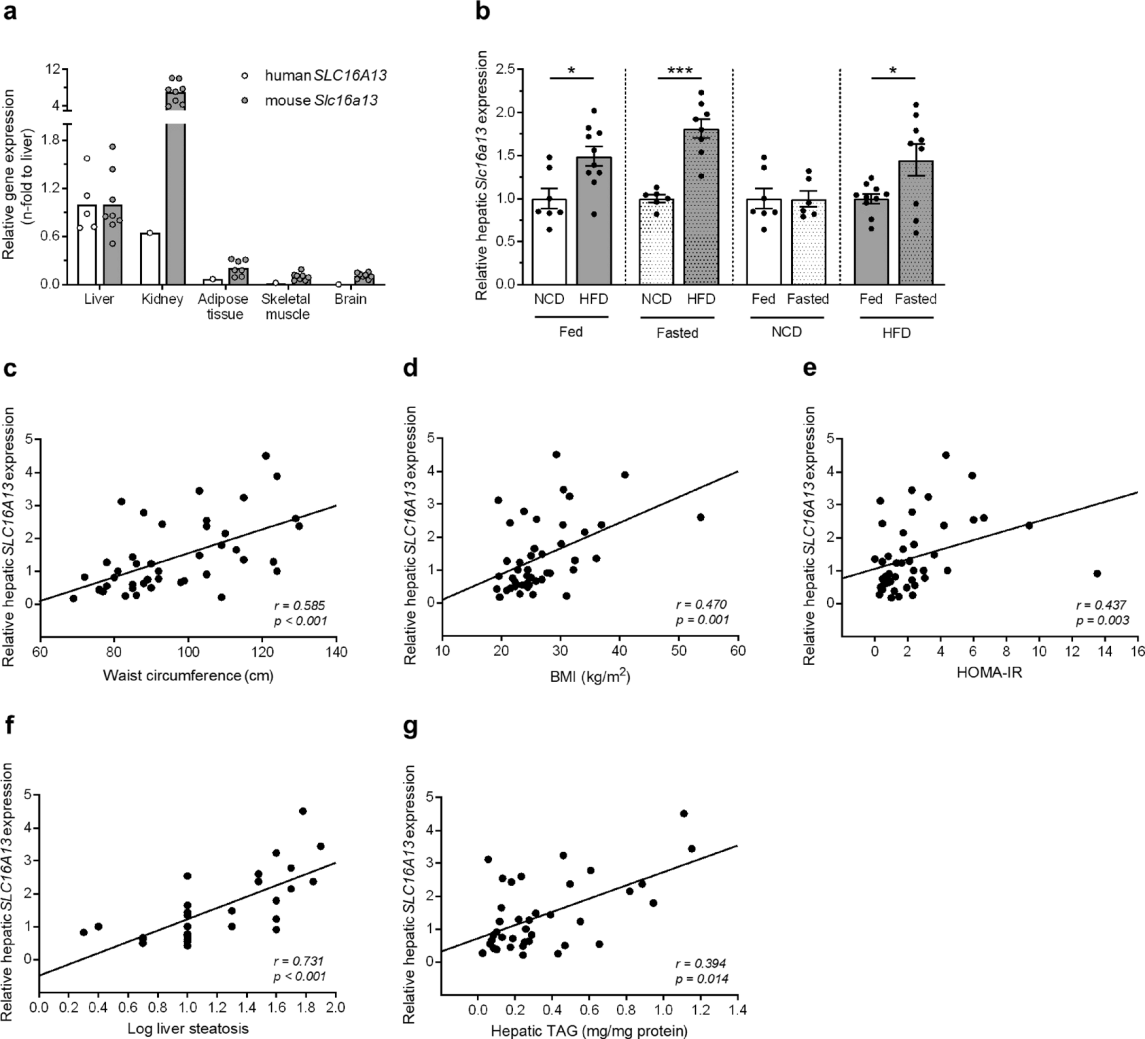
*SLC16A13* expression in humans and mice. **a** *SLC16A13* mRNA expression in one human sampler relative to mRNA expression in the liver (*n* = 5). *Slc16a13* mRNA expression in mice fed normal-chow diet (*n* = 7–8) relative to mRNA expression in the liver. **b** Relative hepatic *Slc16a13* mRNA expression in C57BL/6J mice fed with NCD or HFD for 15 weeks. Animals are fed (*n* = 7–10 per group) or fasted for 16 h (*n* = 6–9 per group). **a**, **b** Bars represent means ± SEM. *p < 0.05, **p < 0.01 determined using two-tailed unpaired Student’s *t*-test. **c**–**g** Correlation of relative hepatic *SLC16A13* mRNA expression and adiposity-related parameters in humans. Linear regression curve is shown. **c** Waist circumference, **d** BMI (Body Mass Index), **e** HOMA-IR (Homeostatic Model Assessment for Insulin Resistance), **f** Logarithm liver steatosis, **g** Hepatic TAG (triacyl**g**lycerol). *r* = Spearman-Rho correlation coefficient.

*SLC16A13/Slc16a13* is expressed in human and mouse liver and only marginally expressed in other metabolically active organs such as adipose tissue, brain, and skeletal muscle (Fig. 1a and Supplementary Fig. 1a). This expression pattern is similar to published human data showing the highest abundance of *SLC16A13* expression in liver tissue^3^. Of note, in mice but not in humans, *Slc16a13* expression is highest in the kidney. However, in our study, we focused on the role of SLC16A13 in the liver for three reasons: First, the overall aim of the study is to investigate the potential role of SLC16A13 in human metabolic disease; second, human *SLC16A13* expression is the highest in the liver, and third, the liver is crucial in glucose and lipid metabolism. Because the human *SLC16A13* polymorphism is associated with T2D, we speculated that *SLC16A13* expression and/or protein function might also be dysregulated in the context of metabolic disease independent of the known polymorphism. To address this point, we analyzed *Slc16a13* gene expression under conditions of obesity. In diet-induced obese mice, *Slc16a13* expression is differentially regulated in the liver (Fig. 1b). Hepatic *Slc16a13* expression was up to 1.8-fold higher in obese animals compared to mice fed a normal-chow diet (NCD), an effect that was observed both under fed and fasted conditions. The observed increase by high-fat diet strongly suggests the possibility that the SLC16A13 transporter is induced by an excess of the nutritional substrate during periods of high caloric supply. Strikingly, we observed a fasting-feeding difference in *Slc16a13* gene expression in HFD-fed mice, while there was no such nutritional dependence in NCD-fed mice, demonstrating a diet-dependent difference in the molecular regulation of this gene. Diet-induced obesity may lead to dysregulation of *Slc16a13* expression, making it impossible to regulate it normally during fasting. In contrast, *Slc16a13* expression in other tissues than the liver is not or only marginally upregulated in the tested dietary conditions (Supplementary Fig. 1b), suggesting a key role of the liver in Slc16a13-mediated metabolic dysregulation. To further investigate the potential role of the transporter in human metabolic disease, we analyzed *SLC16A13* gene expression in human liver samples with different stages of NAFLD and insulin resistance. Liver specimens were obtained from 45 human patients after partial hepatectomy; regions of the liver without pathological findings were biopsied and were processed for gene expression analysis. Physical and clinical parameters have been recorded for all patients and tissue NAFLD-activity score was determined by a sample-masked pathologist (Supplementary Table 1). Hepatic *SLC16A13* gene expression significantly correlated with waist circumference (Fig. 1c), BMI (Fig. 1d), HOMA-IR (Fig. 1e), and histologically assessed liver steatosis (Fig. 1f) as well as with quantitatively measured liver TAG content (Fig. 1g). Interestingly, the highest positive correlation (*r* = 0.731) was observed for liver steatosis (Fig. 1f). The association with liver steatosis remained significant after adjustment for main confounders (Supplementary Table 2). These human data provide evidence for an association between *SLC16A13* gene expression and the development of adiposity, fatty liver, and insulin resistance independent of the described human polymorphism. Understanding the link between increased *SLC16A13* expression and these metabolic phenotypes will help to uncover its potential contribution to T2D development.

### Slc16a13 transports l-lactate across the plasma membrane

**Fig. 2:**
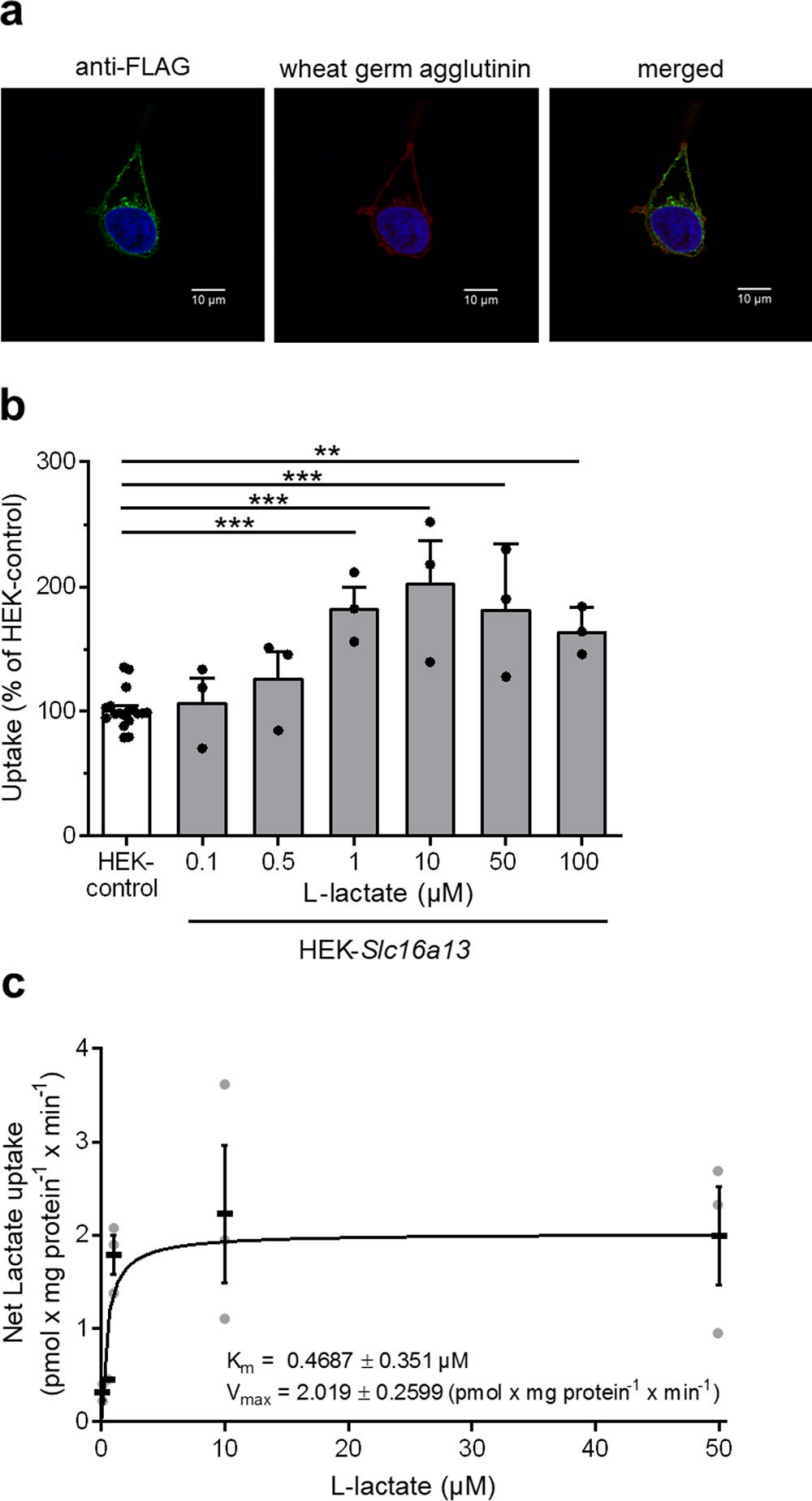
Mouse Slc16a13 cellular localization and transport activity. **a** Immunofluorescence co-staining of the plasma membrane marker wheat germ agglutinin and FLAG-tagged Slc16a13 in HEK-*Slc16a13-FLAG* cells. One representative staining is shown. **b** l-lactate uptake into HEK-control and HEK-*Slc16a13* cells (*n* = 3 for each concentration). Bars represent means ± SEM. **p < 0.01, ***p < 0.001 determined using one-way ANOVA followed by Dunnett’s multiple comparison test. **c** Net l-lactate uptake as the difference between the uptake into HEK-control and HEK-*Slc16a13* cells. Nonlinear regression curve and means ± SEM are displayed.

As an orphan transporter, the precise function and role of SLC16A13 remain to be determined. Knowledge of substrate specificity and localization would assist in the development of hypotheses regarding its role in metabolic control. To characterize cellular localization and substrate specificity of the Slc16a13 transporter, we stably transfected HEK293 cells with a vector containing *Slc16a13* cDNA. Human SLC16A13 was previously described to be located at the Golgi apparatus but has not been detected at the plasma membrane^3^. Here, we could confirm that mouse Slc16a13 localizes to the Golgi apparatus (Supplementary Fig. 2), but importantly, based on immunofluorescence co-staining with the plasma membrane marker wheat germ agglutinin, we observed that mouse Slc16a13 also localizes to the plasma membrane (Fig. 2a and Supplementary Fig. 2). To further characterize the biology of the Slc16a13 transporter, substrate uptake experiments were performed with the HEK-*Slc16a13* cell line. l-lactate is a prominent monocarboxylate transported by other SLC16 family members. At l-lactate concentrations ranging from 1 to 100 µM, Slc16a13-dependent l-lactate transport was increased up to 2-fold in HEK-*Slc16a13* compared to the HEK-control cell line (Fig. 2b). The net uptake of l-lactate, defined by the difference in uptake between HEK-*Slc16a13* and HEK-control cells, was used to calculate the *K*_m_ (0.47 ± 0.35 µM) and *V*_max_ values (2.02 ± 0.26 pmol × mg protein^−1^ × min^−1^) (Fig. 2c). These results suggest that Slc16a13 is a high-affinity and low-capacity transporter for l-lactate.

### Generation of *Slc16a13* knockout mice

**Fig. 3:**
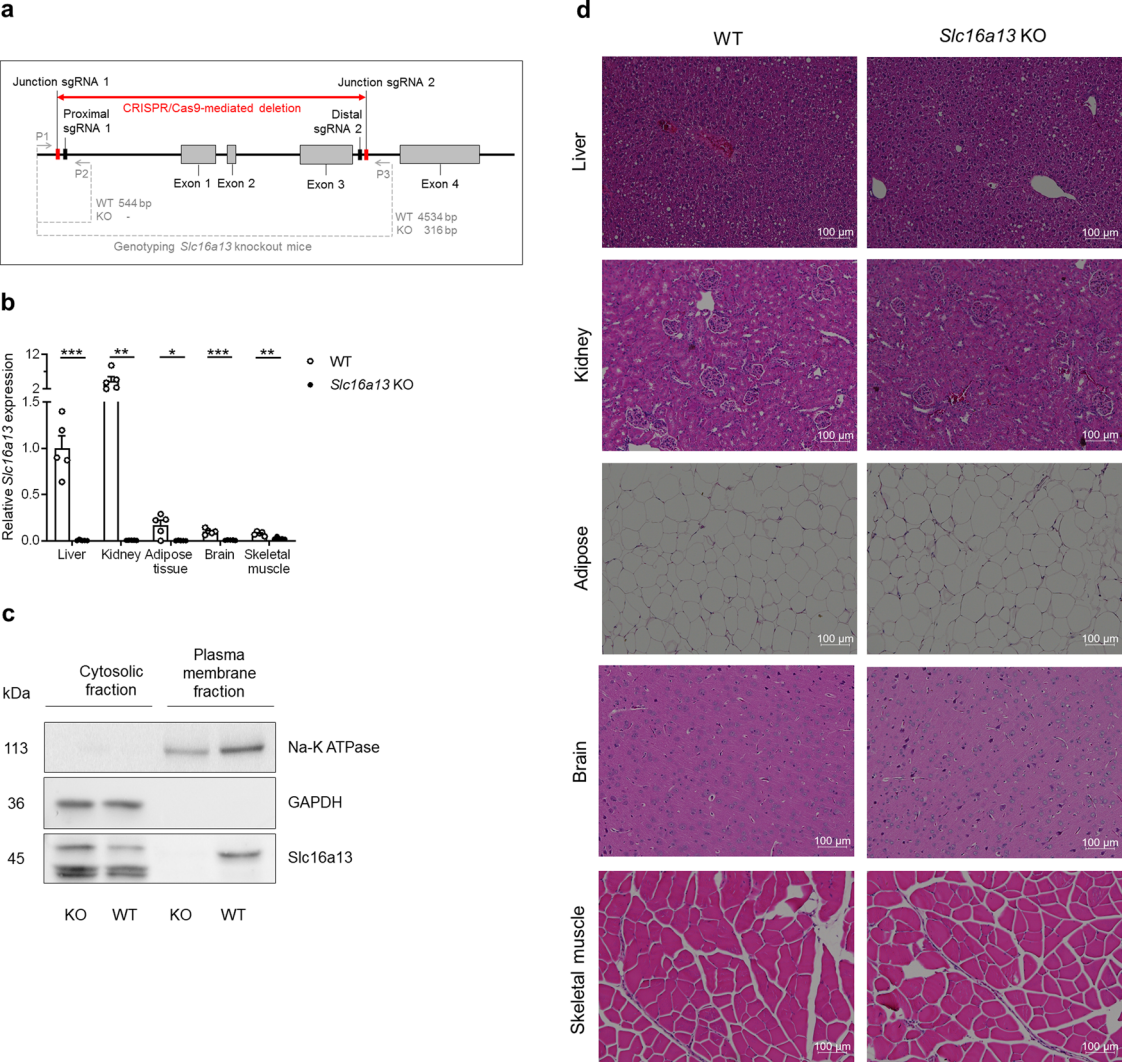
Generation of *Slc16a13* knockout mice using CRISPR/Cas9 technology. **a** Deletion of 4.2 kb including 1.8 kb of the proximal promotor region, exon 1, exon 2, and exon 3 of the mouse *Slc16a13* gene as indicated by the red arrow. To distinguish between the wild-type (WT) and *Slc16a13* knockout (KO) allele on the DNA level, a forward primer (P1) binding upstream of junction 1 is used in combination with the two reverse primer P2 binding within and P3 binding downstream the deleted region as indicated by the gray arrows. mRNA expression levels in WT and KO tissue are analyzed by qRT-PCR primer binding at the exon-exon junctions of exon 1/2 and exon 2/3 (not displayed in the figure). **b** *Slc16a13* mRNA expression in different organs of *Slc16a13* KO mice relative to WT (*n* = 4–5 each group). Relative expression calculated as fold change to the liver. Bars represent means ± SEM. *p < 0.05, **p < 0.01, ***p < 0.001 determined using two-tailed unpaired Student’s *t*-test. **c** Western blot analysis of cytosolic and plasma membrane fraction extracted from liver lysates of *Slc16a13* KO and WT mice. **d** Histological comparison of *Slc16a13* KO mice and WT controls at a mean age of 20 weeks. Representative H&E stainings of the liver, kidney (cortex), white adipose tissue, brain (cortex), and skeletal muscle (quadriceps) at ×10 magnification.

Both human and mouse expression data (Fig. 1b–g) do not indicate whether SLC16A13 deregulation is causal or just a consequence of metabolic alterations in the context of obesity. Since SLC16A13 transports l-lactate across the plasma membrane, we hypothesize that the transporter causally contributes to the development of NAFLD and insulin resistance through changes in hepatic monocarboxylate transport, affecting cellular energy metabolism, hepatic lipid content, and insulin sensitivity. To test this hypothesis in an in vivo model, we generated constitutive *Slc16a13* knockout mice using CRISPR/Cas9 technology (Fig. 3a). The homozygous knockout of *Slc16a13* generates viable offspring in Mendelian ratios. To exclude that CRISPR/Cas9-mediated *Slc16a13* gene editing targets other genes, off-target analysis was performed in the G1 generation and mRNA expression of *Slc16a11* and *Bcl6b*, located adjacently to *Slc16a13* on mouse chromosome 11, was analyzed in the G3 generation (Supplementary Fig. 3). Efficient deletion of *Slc16a13* was confirmed by gene expression analysis of multiple tissues, with the liver having <1% of remaining *Slc16a13* expression compared to wild-type mice (Fig. 3b), and by western blot analysis of wild-type and knockout liver lysates that manifests Slc16a13 protein in the plasma membrane of wild-type, but not of knockout livers (Fig. 3c and Supplementary Fig. 12a). Histological comparison of liver, kidney, white adipose tissue, brain, and skeletal muscle did not reveal any morphological abnormalities due to *Slc16a13* deletion in knockout mice (Fig. 3d).

### *Slc16a13* knockout mice show mild metabolic changes

**Fig. 4:**
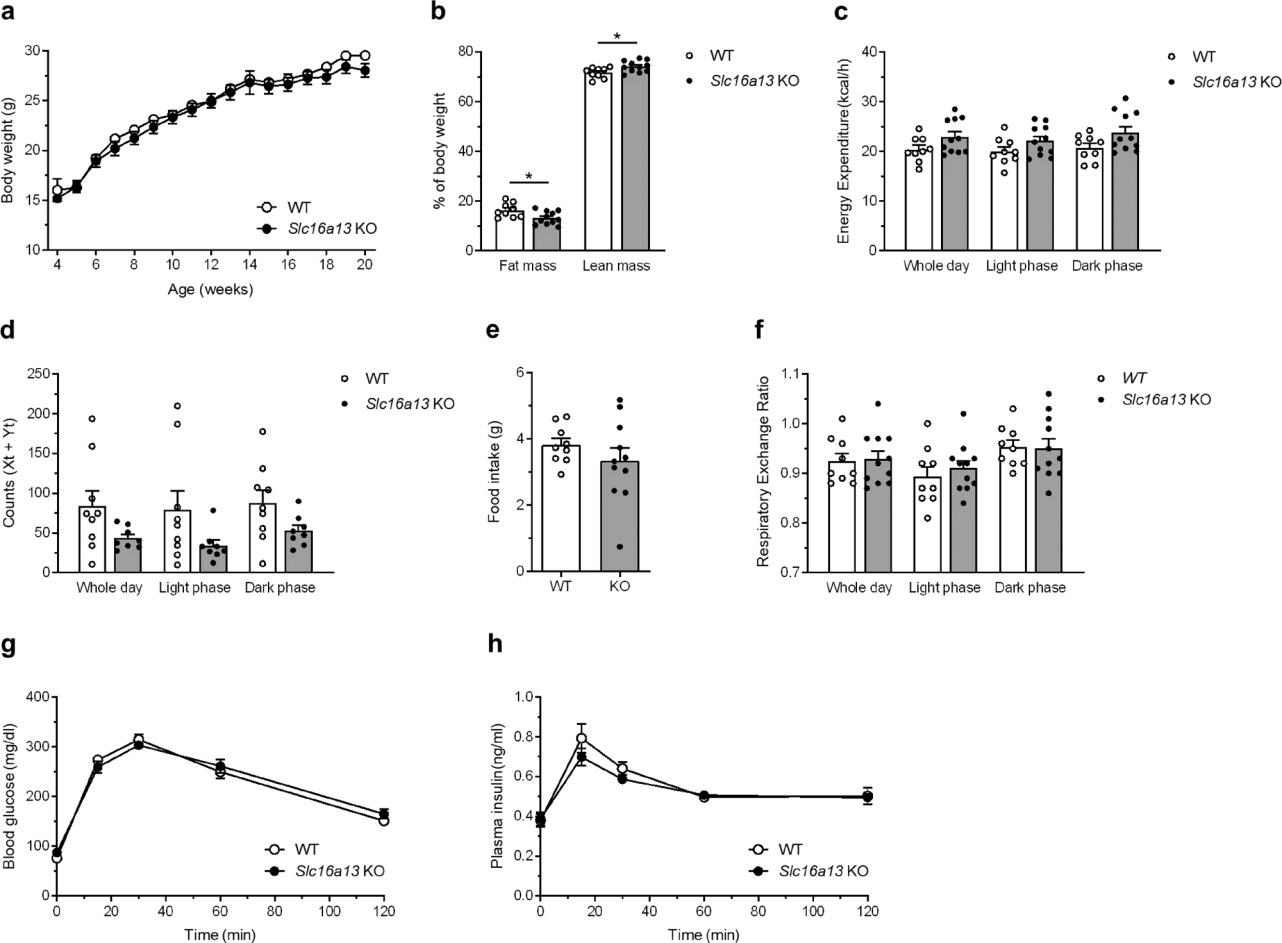
Metabolic phenotype of NCD-fed *Slc16a13* knockout mice. **a** Body weight of WT and *Slc16a13* KO mice over a time course of 18 weeks. **b**–**f** Body composition, energy expenditure, locomotor activity, food intake, and respiratory exchange ratio (RER) of WT, and *Slc16a13 KO* mice at an age of 14 weeks. **g**, **h** Blood glucose levels and plasma insulin levels of WT and *Slc16a13* KO mice after oral glucose administration at an age of 12 weeks. *Slc16a13* WT (*n* = 9), *Slc16a13* KO (*n* = 8–11). **a**, **g**, **h** Means ± SEM are displayed. **b**–**f** Bars represent means ± SEM. *p < 0.05 determined using two-tailed unpaired Student’s *t*-test.

**Fig. 5:**
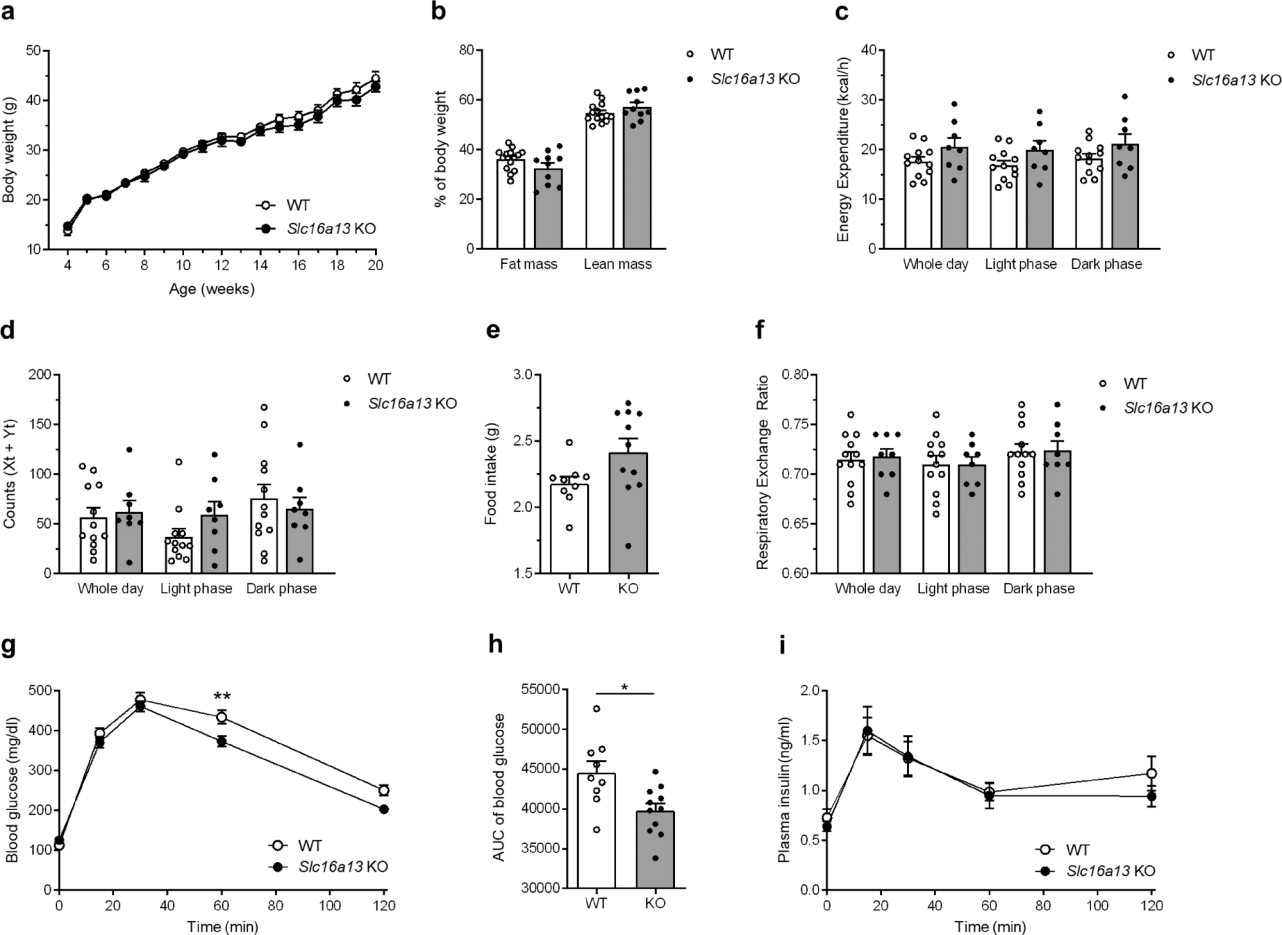
Metabolic phenotype of HFD-fed *Slc16a13* knockout mice. **a** Body weight of WT and *Slc16a13* KO mice over a time course of 18 weeks. **b**–**f** Body composition, energy expenditure, locomotor activity, food intake, and respiratory exchange ratio (RER) of WT, and *Slc16a13* KO mice at an age of 14 weeks. **g**–**i** Blood glucose levels (**g**), related area under the curve (AUC) (**h**) and plasma insulin levels (**i**) of WT and *Slc16a13* KO mice after oral glucose administration at an age of 12 weeks. *Slc16a13* WT (*n* = 9–14), *Slc16a13* KO (*n* = 8–11). **a**, **g**, **i** Means ± SEM are displayed. **b**–**f**, **h** Bars represent means ± SEM. **p < 0.01 determined using two-way ANOVA followed by Sidak’s multiple comparison test (**g**) and *p < 0.05 determined using two-tailed unpaired Student’s *t*-test (**h**).

*Slc16a13* knockout and wild-type control mice were fed either normal-chow (NCD) or high-fat diet (HFD) for 15 weeks. *Slc16a13* knockout mice showed a body weight equal to wild-type mice when fed NCD (Fig. 4a). Interestingly, the deletion of *Slc16a13* led to a reduction in fat mass up to 20% compared to wild-type mice (Fig. 4b). However, energy expenditure, locomotor activity, food intake, and respiratory exchange ratio (RER) were not different between the genotypes at 9 weeks of NCD (Fig. 4c–f, respectively). Furthermore, deletion of *Slc16a13* did not affect glucose metabolism as indicated by similar glucose tolerance in knockout and wild-type mice at 7 weeks of NCD (Fig. 4g). Plasma insulin levels were unaltered after glucose administration (Fig. 4h), excluding a compensatory hyperinsulinemia to maintain blood glucose levels. In the context of diet-induced obesity, the knockout of *Slc16a13* did not significantly affect body weight, body composition, or energy expenditure (Fig. 5a–c), suggesting obesity might mask the Slc16a13-dependent effect on body composition seen in NCD-fed mice. Locomotor activity, food intake, and respiratory exchange ratio (RER) were also unaltered at 9 weeks of HFD (Fig. 5d–f). In contrast, already after 7 weeks of HFD-feeding, loss of Slc16a13 function improved glucose tolerance, demonstrated by significantly decreased blood glucose levels in *Slc16a13* knockout mice 60 min after oral glucose administration and significantly lower area under the curve (AUC) (Fig. 5g, h), while insulin levels were unchanged between the genotypes (Fig. 5i). These data suggest a modulatory role of SLC16A13 in glucose metabolism in the context of diet-induced obesity.

### *Slc16a13* knockout attenuates insulin resistance in HFD-fed mice

**Fig. 6:**
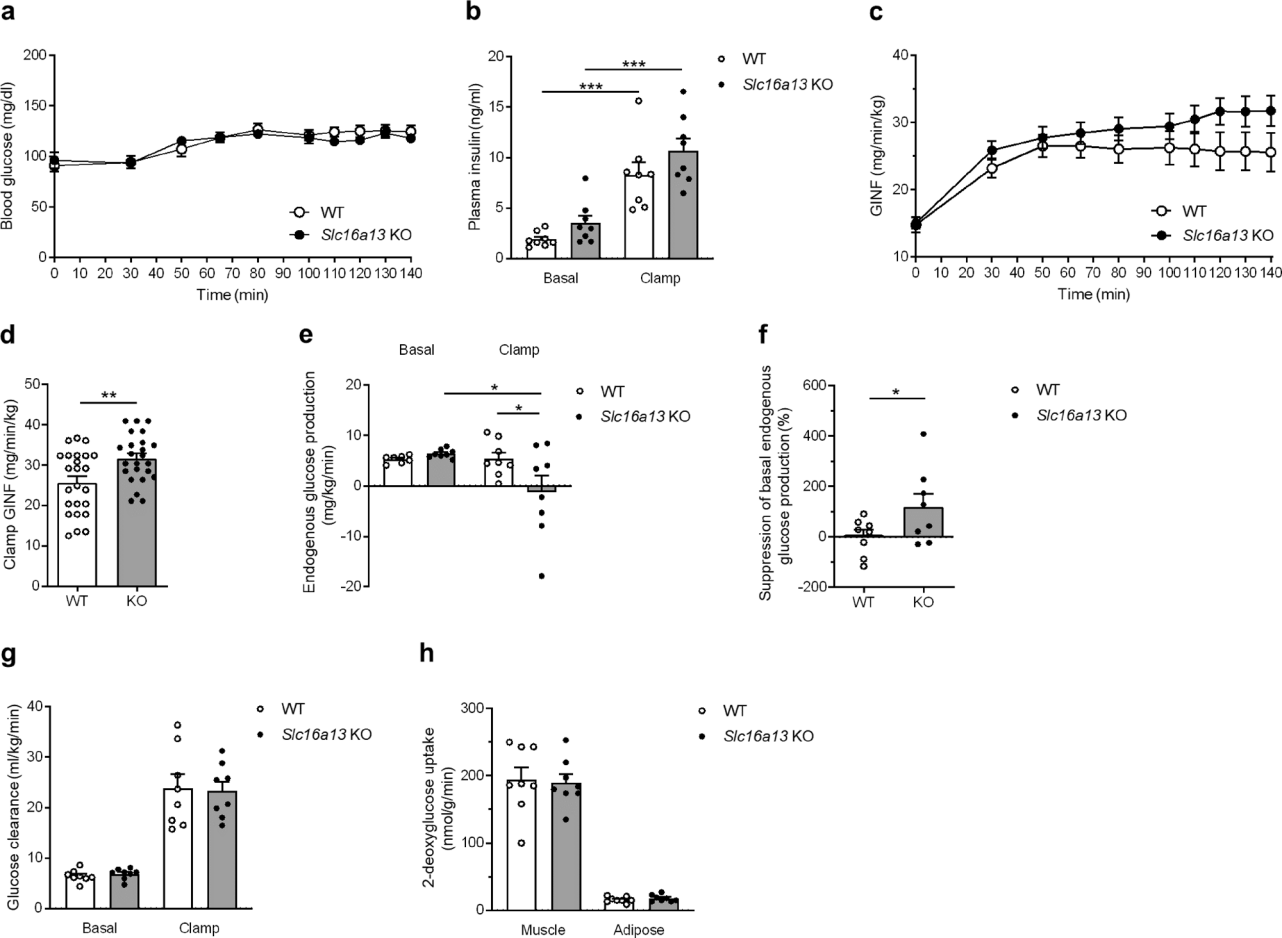
Glucose metabolism of *Slc16a13* knockout mice at 15 weeks of HFD. **a** Plasma glucose concentration during HE clamp. **b** Basal and clamp plasma insulin levels. **c**, **d** Glucose-infusion rate during HE clamp and in the clamped state (120–140 min) in WT and *Slc16a13* KO mice. All three steady-state data points per mouse are shown (**d**). **e**, **f** Basal and clamp hepatic endogenous glucose production and suppression of basal endogenous glucose production in WT and *Slc16a13* KO mice. **g** Plasma glucose clearance in WT and *Slc16a13* KO mice. **h** 2-deoxyglucose uptake in quadriceps muscle and epigonadal white adipose tissue. *Slc16a13* WT (*n* = 8), *Slc16a13* KO (*n* = 8). **a**, **c** Means ± SEM are displayed. **b**, **d**–**h** Bars represent means ± SEM. *p < 0.05, **p < 0.01, ***p < 0.001 determined using two-tailed unpaired Student’s *t*-test, except for **e**, **f** *p < 0.05 determined using one-tailed unpaired Student’s *t*-test.

Since an oral glucose tolerance test is only a rough marker of insulin sensitivity, we investigated whole body and tissue-specific insulin action by hyperinsulinemic–euglycemic (HE) clamp after 15 weeks of HFD-feeding. During HE clamp, blood glucose and insulin levels did not differ significantly between *Slc16a13* knockout and wild-type mice (Fig. 6a, b). Consistent with the lower blood glucose levels seen in the oral glucose tolerance test (Fig. 5g, h), the glucose-infusion (GINF) rate was higher in *Slc16a13* knockout mice (Fig. 6c) and significantly increased by 20% in the clamp steady-state (Fig. 6d). To address the tissue-specific contributions to the improved insulin sensitivity, we determined endogenous hepatic glucose production and glucose uptake into peripheral organs. Insulin-mediated suppression of endogenous glucose production, a reflection of hepatic insulin sensitivity, was markedly better in *Slc16a13* knockout mice than wild-type mice (Fig. 6e, f), demonstrating attenuation of HFD-induced hepatic insulin resistance. Accordingly, insulin downstream signaling was improved in *Slc16a13* knockout mice as addressed by Akt phosphorylation in liver tissue (Supplementary Fig. 4a), whereas liver glycogen storage was unaltered (Supplementary Fig. 4b). Insulin-stimulated glucose clearance was not different between *Slc16a13* knockout and wild-type mice, demonstrating no effect on peripheral insulin sensitivity (Fig. 6g). Consistent with this, 2-deoxyglucose uptake into skeletal muscle and adipose tissue was not affected by genotype (Fig. 6h). Therefore, attenuation of insulin resistance in obese *Slc16a13* knockout mice could be attributed to improved hepatic insulin sensitivity.

### HFD-fed *Slc16a13* knockout mice are protected from hepatic lipid accumulation

**Fig. 7:**
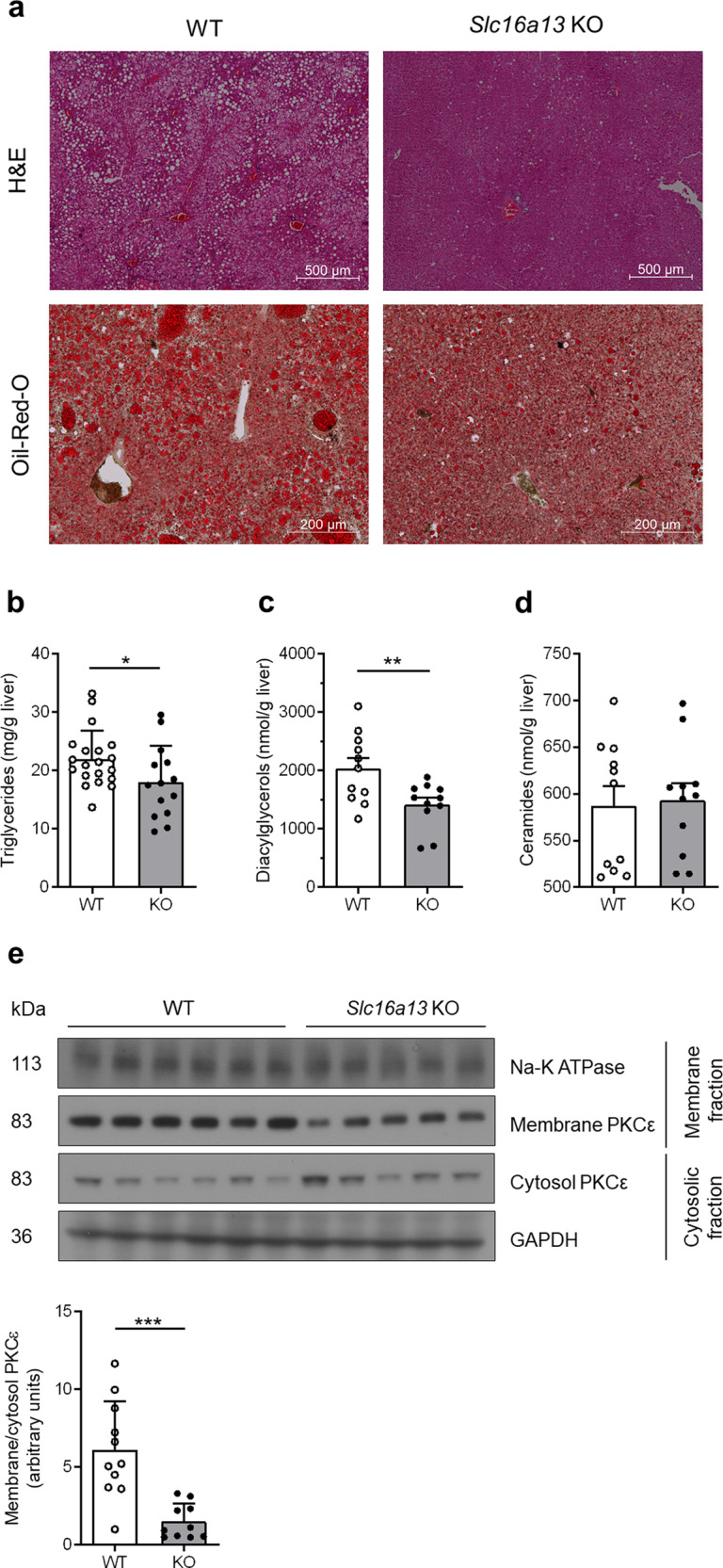
Liver lipids of *Slc16a13* knockout mice at 15 weeks of HFD. **a** Representative H&E and Oil-Red-O stainings of WT and *Slc16a13* KO mice. **b** Hepatic triglycerides in WT (*n* = 20) and *Slc16a13* KO mice (*n* = 14). Two cohorts of mice have been analyzed. **c**, **d** Hepatic diacylglycerol and ceramide level in WT and *Slc16a13* KO mice (*n* = 11 each group). **e** PKCε protein expression determined by western blot shown for WT (*n* = 6) and *Slc16a13* KO mice (*n* = 5). PKCε translocation assessed as the ratio of membrane to cytosol PKCε in WT and *Slc16a13* KO mice (*n* = 11 each group). Bars represent means ± SEM. **p < 0.05, **p < 0.01, ***p < 0.001 determined using two-tailed unpaired Student’s *t*-test.

Ectopic lipid accumulation in the liver causes fatty liver disease and insulin resistance^19,20^. In accordance with improved insulin sensitivity, H&E and Oil-Red-O staining of liver sections revealed less lipid droplets in *Slc16a13* knockout mice compared to controls after 15 weeks of HFD-feeding (Fig. 7a). Hepatic lipid accumulation includes many species but mainly triglycerides, which seem to be metabolically inert. In contrast, diacylglycerols (DAGs) and ceramides have been shown to be bioactive mediators of insulin resistance in mice and humans^21,22^. Interestingly, the hepatic content in triglycerides, as well as diacylglycerols, was significantly reduced in *Slc16a13* knockout mice (Fig. 7b, c), whereas that of ceramides was unaltered (Fig. 7d). DAGs are known to mediate insulin resistance via activation of atypical protein kinases, such as PKCε in the liver. Once activated, cytosolic PKC converts into an active membrane-associated form that phosphorylates the insulin receptor on threonine 1160 (in mouse threonine 1150), which, in turn, inhibits the receptor intrinsic tyrosine kinase activity^21,23^. Compared to wild-type mice, the amount of membrane-associated PKCε was reduced more than 3-fold in the livers of *Slc16a13* knockout mice (Fig. 7e and Supplementary Fig. 12b). Together, our data suggest that *Slc16a13* deletion attenuates the ectopic deposition of lipids in the liver, leading to a decrease in hepatic DAG content, lower PKCε activation, and, in turn, reduces diet-induced hepatic insulin resistance. In this model, our data also dissociate hepatic ceramide content from changes in hepatic insulin sensitivity.

### Reduced hepatic lipid accumulation is caused by increased mitochondrial respiration in *Slc16a13* knockout mice

**Fig. 8:**
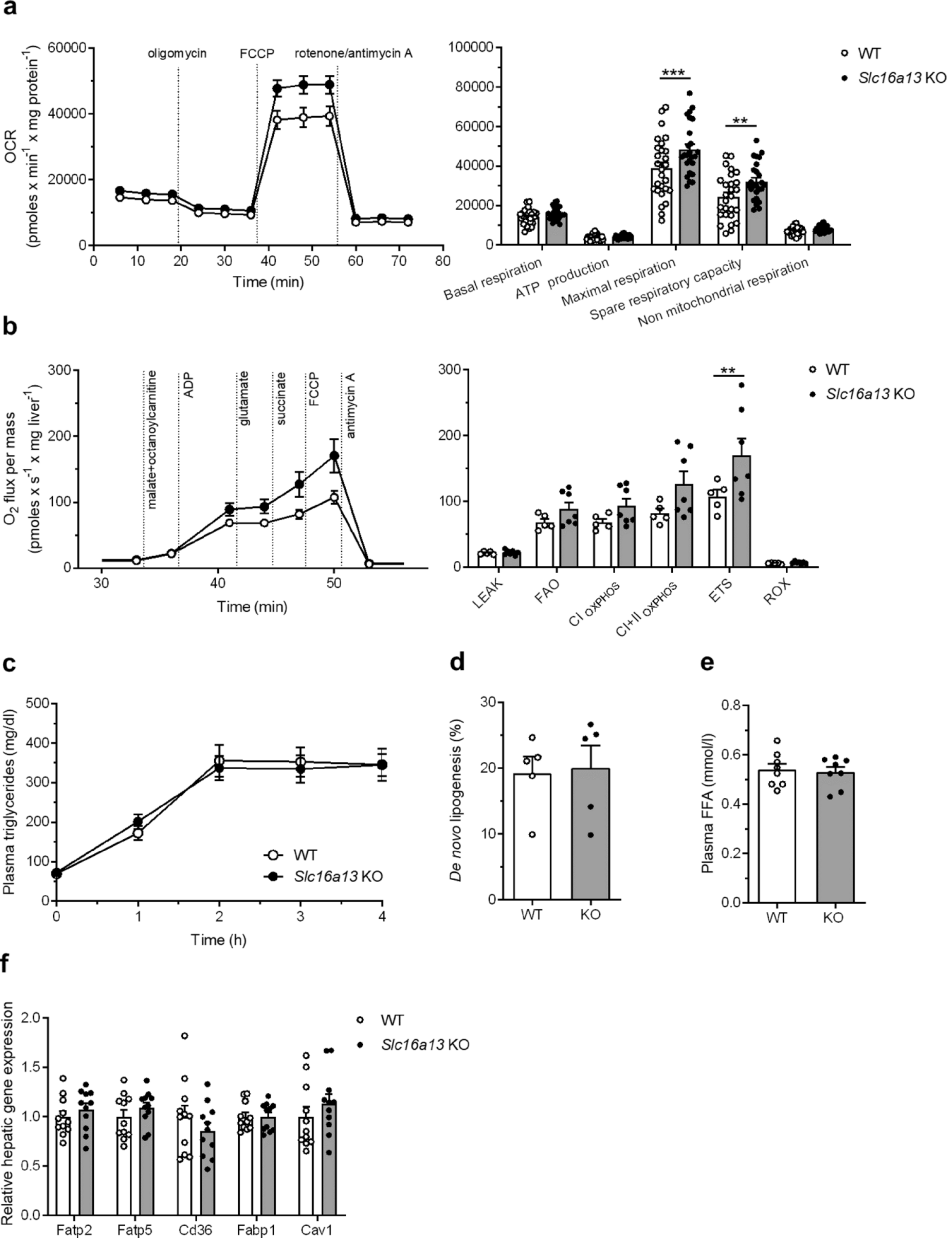
Mechanisms of reduced hepatic lipid accumulation in *Slc16a13* knockout mice. **a** Mitochondrial respiration of hepatocytes isolated from *Slc16a13* WT (*n* = 27) and *Slc16a13* KO (*n* = 25) mice at a mean age of 20 weeks, both groups females fed NCD. Time course and calculated parameters of oxygen consumption rate (OCR) during mitochondrial stress test performed in the Seahorse Analyzer XF96. **b** Mitochondrial respiration of liver tissue isolated from 20 weeks old HFD-fed *Slc16a13* WT (*n* = 5) and *Slc16a13* KO (*n* = 7) mice. Time course and calculated parameters of oxygen flux during high-resolution respirometry performed in the Oroboros Oxygraph-2k. LEAK = proton leak, ion leak, and slip compensatory state; FAO = fatty acid oxidation; OXPHOS = oxidative phosphorylation; CI_OXPHOS_ = complex I-related oxidative phosphorylation capacity; CI + II_OXPHOS_ = complex I and II-related oxidative phosphorylation capacity; ETS = electron transfer system; ROX = residual oxygen consumption. **c** Plasma triglyceride levels after intraperitoneal injection of the LPL-inhibitor Poloxamer-407 at an age of 18 weeks. **d** Hepatic de novo lipogenesis in WT and *Slc16a13* KO mice at 20 weeks of age. **e** Plasma level of free-fatty acids (FFA) in a fasted state. **f** Relative hepatic mRNA expression of fatty acid transporters. *Slc16a13* WT (*n* = 5–11), *Slc16a13* KO (*n* = 5–11). **a**–**c** Means ± SEM are displayed. **a**, **b**, **d**–**f** Bars represent means ± SEM. **p < 0.01, ***p < 0.001 determined using two-way ANOVA followed by Sidak’s multiple comparison test.

Hepatic lipid content is regulated by four mechanisms: β-oxidation, *de novo* lipogenesis, lipid export as VLDL particles, and uptake of circulating fatty acids^20,24^. Loss of SLC16A13 function might counteract one of these mechanisms in the context of diet-induced obesity. To identify the underlying mechanism for reduced fat content in the livers of *Slc16a13* knockout mice, we measured oxygen consumption of *Slc16a13* knockout and wild-type hepatocytes isolated from NCD-fed females at a mean age of 20 weeks. During Seahorse XF Cell Mito Stress Test using the Seahorse Analyzer, cells were treated with the ATP synthase (complex V) inhibitor oligomycin, the mitochondrial uncoupler FCCP and rotenone/antimycin A, inhibitors of complexes I and III of the mitochondrial respiratory chain, respectively. Deletion of *Slc16a13* was associated with higher mitochondrial respiration, indicated by a higher oxygen consumption rate (OCR) and a significant 25% increase in maximal respiration (Fig. 8a). To investigate this observation in more detail, we performed high-resolution respirometry in the Oroboros O2k oxygraph using the substrate-uncoupler-inhibitor titration (SUIT) protocol on liver tissue isolated from mice fed HFD for 15 weeks. The application of the substrates malate, octanoylcarnitine, ADP, glutamate, and succinate, the uncoupler FCCP, and the inhibitor antimycin A allows the analysis of β-oxidation-linked mitochondrial respiration. Mitochondrial function measured as O_2_ flux per mass was increased in livers of *Slc16a13* knockout mice and consistent with the Seahorse data showing a 60% greater maximum capacity of the electron transport chain (ETS) in knockout livers (Fig. 8b). Furthermore, respiratory control ratios calculated from respirometry data indicate that mitochondria in Slc16a13 deficient liver become more effective at generating ATP from fatty acids (Supplementary Fig. 5). The fatty acid OXPHOS coupling control factor is significantly increased with loss of Slc16a13 and indicates that this is primarily due to higher OXPHOS capacity and not caused by reduction of the respiratory LEAK, suggesting improved control and/or capacity of the ADP phosphorylation system regarding the respiratory flux after fatty acid utilization (Supplementary Fig. 5a). In line with this, the respiratory control ratio is increased and the LEAK control ratio is decreased in livers of *Slc16a13* knockout mice (Supplementary Fig. 5b, c, respectively). These ratios suggest a more effective ATP synthesis from fatty acids, what may be the reason for increased fatty acid oxidation. To investigate whether other mechanisms might contribute to the reduced hepatic lipid accumulation, VLDL export, de novo lipogenesis, and FFA uptake were analyzed in these mice. Plasma triglyceride levels addressing hepatic VLDL production, assessed by injection of the lipoprotein lipase (LPL)-inhibiting detergent Poloxamer-407 in fasted mice at 13 weeks of HFD, were unaltered between *Slc16a13* knockout and wild-type mice (Fig. 8c). Hepatic de novo lipogenesis, assessed by application of deuterated water, and plasma non-esterified fatty acid concentrations did not differ between the genotypes at 15 weeks of HFD (Fig. 8d, e, respectively), and we observed no difference at the mRNA level of hepatic fatty acid transporters (Fig. 8f). Together, our data suggest that increased mitochondrial β-oxidation is the main mechanism protecting against fatty liver development in *Slc16a13* knockout mice.

### Deletion of *Slc16a13* increases mitochondrial function by AMPK activation

**Fig. 9:**
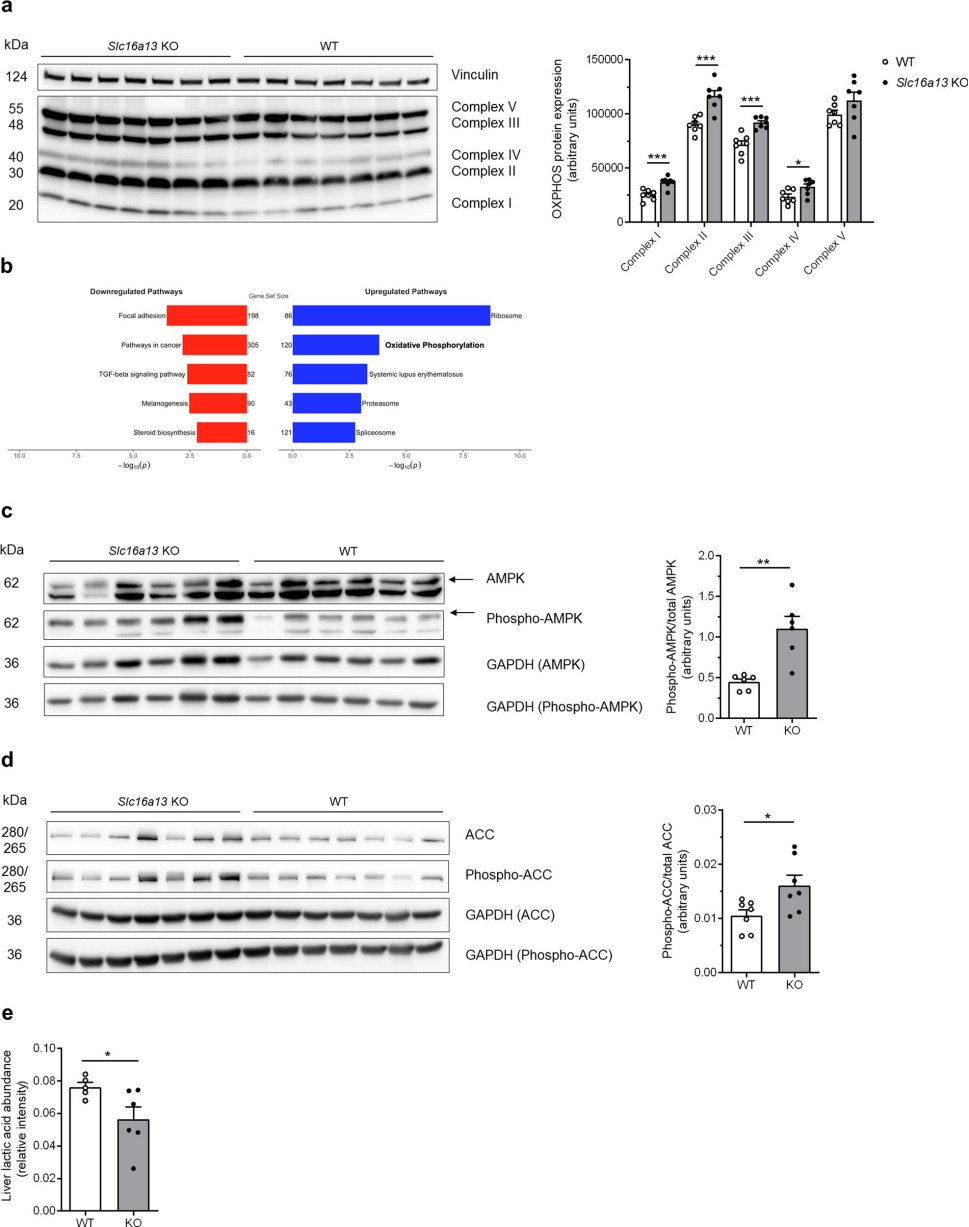
Molecular insight into increased mitochondrial respiration in *Slc16a13* knockout mice at 15 weeks of HFD. **a** Hepatic protein expression of respiratory chain complexes I–V determined by western blot. **b** Gene set enrichment analysis of liver transcriptomic data from WT and *Slc16a13* KO mice. **c** AMPK phosphorylation as a ratio of phospho-AMPK/total AMPK determined by western blot. **d** ACC phosphorylation as ratio of phospho-ACC/total ACC determined by western blot. *Slc16a13* WT (*n* = 6–7), *Slc16a13* KO (*n* = 6–7). **e** Relative lactic acid levels determined by untargeted metabolomics in the livers of WT (*n* = 5) and *Slc16a13* KO mice (*n* = 6). **a**, **c**–**e** Bars represent means ± SEM. *p < 0.05, **p < 0.01, ***p < 0.001 determined using two-tailed unpaired Student’s *t*-test, Welch correction for unequal variance was used in **e**.

To investigate mitochondrial function at the molecular level, we performed western blot analysis on liver lysates and found the protein expression of respiratory chain complexes to be significantly higher in *Slc16a13* knockout mice compared to wild-type littermates after 15 weeks of HFD-feeding (Fig. 9a and Supplementary Fig. 12c). This is consistent with transcriptomic data showing significantly upregulated oxidative phosphorylation in knockout compared to wild-type liver as determined by gene set enrichment analysis (Fig. 9b). In contrast, neither OXPHOS protein levels nor gene set enrichment analysis indicated deregulated oxidative phosphorylation in skeletal muscle between the two genotypes (Supplementary Fig. 6a, b, respectively). Thus, we propose *Slc16a13* deletion in the liver is a key causal factor for the increase in oxidative phosphorylation and finally, the improvement in metabolic phenotype observed in HFD-fed *Slc16a13* knockout mice.

**Fig. 10:**
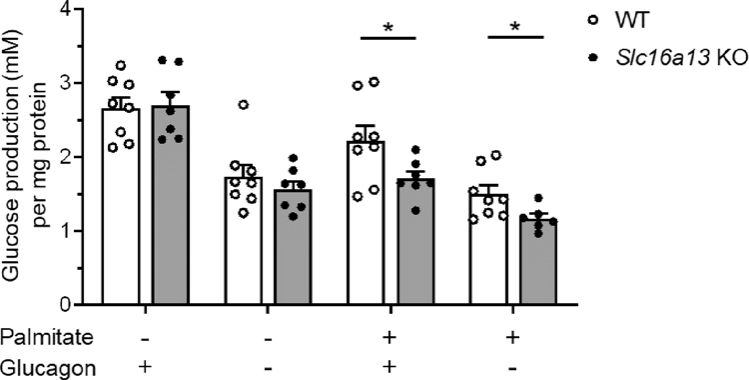
Gluconeogenesis in *Slc16a13* knockout primary hepatocytes. Glucose production of primary hepatocytes isolated from *Slc16a13* WT (*n* = 8) and *Slc16a13* KO (*n* = 7) mice at a mean age of 20 weeks. Hepatocytes were stimulated with 500 µM BSA-coupled palmitate (6:1 molar ratio) or BSA control and 100 nM glucagon or vehicle control as indicated. Cell culture supernatant was collected after 8 h of incubation in glucose-free media containing 20 mM lactate and 2 mM pyruvate. Bars represent means ± SEM. *p < 0.05 determined using two-tailed unpaired Student’s *t*-test.

Given that mitochondrial respiration is increased in *Slc16a13* knockout mice, AMPK, the main sensor of cellular energy status and regulator of mitochondrial homeostasis, could be causative. AMPK is activated by phosphorylation at low energy status, defined by high cellular AMP and low ATP level, and promotes mitochondrial biogenesis and expression of respiratory proteins^25^. Here, protein expression analysis of liver extracts showed a significant increase in the ratio of phospho-AMPK/total AMPK in *Slc16a13* knockout mice (Fig. 9c and Supplementary Fig. 12d). AMPK phosphorylates and thereby inactivates acetyl-CoA carboxylase (ACC), an enzyme that exists in two isoforms, ACC-1 and ACC-2, both highly expressed in the liver^26,27^. AMPK phosphorylation of ACC-1 at serine 212 and ACC-2 at serine 79 reduces fatty acid synthesis and increases fatty acid oxidation through activation of carnitine palmitoyltransferase 1 (CPT1)^28,29^. Importantly, it was shown that both, ACC-1 and ACC-2 regulate fatty acid oxidation, whereas ACC-1 mainly regulates lipogenesis^30^. To further establish the link between AMPK activation and mitochondrial function, we found the ratio of phospho-ACC/total ACC protein was significantly higher in *Slc16a13* knockout liver extracts (Fig. 9d and Supplementary Fig. 12e), in agreement with cellular activation of AMPK. To confirm these results, mouse primary hepatocytes isolated from *Slc16a13* knockout and wild-type mice were treated with the saturated free-fatty acid palmitate to induce insulin resistance mimicking HFD-feeding. Also in these hepatocytes, phosphorylation of AMPK and ACC was significantly increased in cells deficient for Slc16a13 (Supplementary Fig. 7a). In contrast, BSA control-stimulated hepatocytes did not show this difference in protein phosphorylation (Supplementary Fig. 7b), suggesting that an insulin-resistant state induced by palmitate in vitro, or by HFD-feeding in vivo, is mandatory to provoke these changes in the context of Slc16a13 deficiency. Since we showed that Slc16a13 is a plasma membrane l-lactate transporter (Fig. 2a–c), we postulated the energy deficit in the liver of *Slc16a13* knockout mice stems from the reduction in intracellular lactate level^31,32^. Metabolome analysis of the liver was consistent with this hypothesis, showing a significant reduction of l-lactate level by 25% in *Slc16a13* knockout compared to wild-type liver (Fig. 9e). In contrast, monocarboxylates transported by other SLC16 family members did not differ between wild-type and knockout livers (Supplementary Fig. 8a). To finally test the hypothesis that reduced lactate transport in the liver is the primary metabolic impact of Slc16a13 deficiency, intrahepatocellular lactate utilization was analyzed in *Slc16a13* knockout and wild-type primary hepatocytes (Fig. 10). In palmitate-treated hepatocytes, lactate, and pyruvate-driven glucose production were significantly reduced if Slc16a13 is missing. The reason for reduced gluconeogenesis might be reduced Slc16a13-mediated uptake of lactate into hepatocytes and consequently reduced availability of this gluconeogenic substrate. Furthermore, glucose production was significantly decreased in palmitate-treated *Slc16a13* knockout hepatocytes, but not in BSA control-treated hepatocytes, suggesting that Slc16a13 deficiency only has a metabolic impact under conditions of excess dietary nutrients and linked insulin resistance, as seen for AMPK and ACC phosphorylation (Supplementary Fig. 7). Most importantly, this reduced intrahepatocellular lactate availability will not exclusively affect glucose metabolism, but impact the overall energy status of the cell. According to our hypothesis, intracellular energy deficit may drive AMPK activation and further downstream signaling leading to improved insulin sensitivity in diet-induced obese *Slc16a13* knockout mice. Taken together, these results identify a mechanism for SLC16A13 function in energy homeostasis: loss of SLC16A13 reduces l-lactate uptake in the liver, thereby limiting intracellular substrate availability and reducing intracellular energy levels that lead to activation of AMPK, which promotes mitochondrial respiration. Increased β-oxidation attenuates lipid (DAG) accumulation and the development of hepatic insulin resistance in the context of obesity.

## Discussion

*SLC16A13* has been linked to T2D in humans by two independent GWAS^3,17^. However, the biology and function of the SLC16A13 transporter, and whether alterations in *SLC16A13* are a cause or consequence of T2D, is unknown. Here, we describe a causal relationship between SLC16A13 function and the development of insulin resistance and fatty liver, reflecting the phenotype associated with the *SLC16A13* polymorphism in a cohort of Japanese people. Our hypothesized link between intracellular l-lactate availability and hepatic insulin resistance is supported by the characterization of the transporter localization and substrate specificity in vitro, and by the physiological phenotype of our knockout mouse model in vivo.

Importantly, cellular fractionation of mouse liver lysates clearly demonstrated that endogenous Slc16a13 protein localizes to the plasma membrane (Fig. 3c). In addition, Slc16a13 was identified as a plasma membrane transporter for l-lactate using *Slc16a13*-expressing HEK cells (Fig. 2 and Supplementary Fig. 2). Although l-lactate transport into HEK-*Slc16a13* cells was only increased 2-fold compared to the HEK-control cells (Fig. 2b), recently published data on the SLC16A11 transporter also showed a comparably mild change in SLC16A11-dependent pyruvate transport that still may have an effect on T2D risk^6^. Most importantly, high levels of endogenous transport in HEK cells due to high SLC16A1/MCT1 activity may impede the detection of a stronger Slc16a13-dependent effect. While our investigation only addressed mouse Slc16a13, it is important to highlight that human and mouse protein share 87% protein identity and the human transporter may also localize to the plasma membrane and not only intracellular membranes as presented before by Williams et al.^3^. The description of l-lactate as a primary Slc16a13 substrate is supported by an unbiased metabolomics assessment of the liver that showed lactate was reduced in the livers of *Slc16a13* knockout mice, while other monocarboxylates were not (Fig. 9e and Supplementary Fig. 8a). Although lactate levels are reduced by only 25%, which may be partially explained by MCT1-mediated compensation of lactate transport, these data strongly argue for lactate being a major substrate for Slc16a13. However, we did not investigate transport kinetics using additional substrates and, therefore, cannot rule out the possibility that substrates other than l-lactate are physiologically relevant or contribute to the SLC16A13-associated diabetic phenotype. Further studies will clarify if SLC16A13 also transports other substrates.

*SLC16A1* encodes MCT1, which transports l-lactate, pyruvate, and ketone bodies across the plasma membrane^4^, and it is broadly expressed in contrast to *SLC16A13*. MCT1, along with two other prominent SLC16 family members MCT2 and MCT4, catalyzes the proton-linked influx or efflux of lactic acid in various tissues. Lactic acid is a product of glycolytic cells at low oxygen levels and substrate for gluconeogenesis and lipogenesis in the liver, kidney, and adipose tissue as well as for oxidative phosphorylation in the heart, skeletal muscle, and brain^5,33^. Increased lactate production in adipose tissue due to hypoxia^34^ and decreased oxidative capacity in insulin-resistant muscle^35,36^ contribute to increased circulating lactate levels associated with obesity and T2D^37–39^. Plasma lactate levels are unaltered in *Slc16a13* knockout mice (Supplementary Fig. 8b, c), suggesting that loss of Slc16a13 can be at least partially compensated by other monocarboxylate transporters (e.g., MCT1, MCT2, and MCT4) that facilitate plasma lactate uptake into organs, or that the uptake of liver lactate is insufficient to affect plasma levels. Consistent with unaltered plasma lactate concentrations, muscle insulin sensitivity is unchanged in *Slc16a13* knockout mice (Fig. 6h). An increased expression of hepatic monocarboxylate transporter encoding Slc16 genes was observed in the liver and kidney, what may have served compensatory purposes (Supplementary Fig. 9), indirectly supporting the finding that SLC16A13 transports l-lactate (Fig. 2b). Multiple Slc16 genes are dysregulated in the kidney of *Slc16a13* knockout mice, probably due to the higher expression of *Slc16a13* in the kidney compared to the liver (Fig. 1a). Just two Slc16 genes are significantly upregulated in *Slc16a13* knockout liver under conditions of diet-induced obesity: *Slc16a1* and *Slc16a11*. Both genes encode pyruvate transporter and lactate transport is well characterized for SLC16A1/MCT1. Therefore, it is likely that all three transporter share the same substrates: l-lactate and pyruvate. However, it is unlikely that this compensatory upregulation affects the *Slc16a13* knockout phenotype to a major extend. *Slc16a1* haploinsufficient mice are resistant to diet-induced obesity and associated metabolic perturbations^40^, vice versa *Slc16a1* overexpression would rather result in a detrimental metabolic phenotype, supported by the finding of exercise-induced hyperinsulinemia in human patients with promotor-activating mutations in *SLC16A1*^41,42^ and in a transgenic mouse line with beta cell-specific *Slc16a1* overexpression^43^. Although reduced SLC16A11 activity is proposed to induce metabolic changes associated with a higher risk of T2D^6^, contrary data exist^13,14^, and the significance of *Slc16a11* overexpression remains unclear. Furthermore, both, *Slc16a1* and *Slc16a11* show only slight increases in the liver, and although *Slc16a11* is stronger dysregulated in the kidney, overexpression of SLC16 transporter in the kidney is to our knowledge not linked to metabolic phenotypes of improved insulin sensitivity.

Assuming that SLC16A13 transports l-lactate in the liver, the key question is why loss of SLC16A13 function might be (patho)physiologically relevant, if MCT1 and SLC16A11 show the same substrate specificity and at least MCT1-mediated l-lactate transport is able to compensate SLC16A13 loss. One reason might be the distinct capacity and affinity of substrate transport. Here, we describe Slc16a13 as a low-capacity, but high-affinity transporter for l-lactate with a *K*_m_ value of approximately 0.47 µM in HEK293 cells (Fig. 2c). Compared to MCT2 and MCT4, MCT1 is described as intermediate affinity transporter with *K*_m_ values between 3.5 and 4.5 mM^4,33,44,45^ for the human protein, reflecting lower affinity for l-lactate compared to mouse Slc16a13. Human plasma concentrations of l-lactic acid are ~1.0–1.5 mM^46^, whereas plasma concentrations in our C57BL/6N wild-type mice are higher (Supplementary Fig. 8c). Therefore, SLC16A13 may be a lactate transporter that is quickly saturated, but whose high affinity enables to react to minor changes in plasma lactate concentrations in the early stages of metabolic disease. Compared to MCT1, we speculate that SLC16A13 function is more relevant in liver disease and it may be the transporter mediating hepatic l-lactate influx during specific pathophysiological states. This idea is supported by our gene expression analyses: whereas hepatic *Slc16a13* expression is upregulated in the context of diet-induced obesity (Fig. 1b), *Slc16a1* expression was not found to be induced by HFD- compared to NCD-feeding (Supplementary Fig. 10a). In obese mice, the significant increase in *Slc16a1* expression during fasting might reflect a compensatory upregulation in periods of low substrate supply, even though its hepatic expression is not induced by obesity per se. Furthermore, also *Slc16a11* expression differs under distinct dietary conditions and food deprivation (Supplementary Fig. 10b), pointing towards distinct roles of these SLC16 transporters in diet-induced obesity. However, this hypothesis needs further investigation and to date, the distinct roles of both transporters remain unclear. Importantly, transport kinetics of the human SLC16A13 protein would allow the direct comparison of the *K*_m_ values of both transporters.

*Slc16a13* deletion is significantly associated with lower fat mass in NCD-fed (Fig. 4b), but not in HFD-fed mice (Fig. 5b, *p* = 0.13). Potentially, obesity-related increase in fat mass and decrease in energy expenditure mask the effect seen during normal-chow feeding. Although Slc16a13 has no effect on body weight and energy expenditure, and only a mild effect on fat mass in lean mice fed normal chow, loss of Slc16a13 function results in improved glucose metabolism in obese mice fed HFD as determined by oral glucose tolerance test (Fig. 5g, h) and revealed in more detail using HE clamp (Fig. 6). The significantly higher insulin sensitivity of *Slc16a13* knockout mice can exclusively be explained by increased hepatic insulin sensitivity (Fig. 6e, f) since we did not observe any effects on insulin-stimulated peripheral glucose uptake (Fig. 6h). DAG accumulation due to reduced mitochondrial β-oxidation predisposes to the development of insulin resistance and T2D^21,47–49^. Conversely, we observed increased mitochondrial respiration that reduces hepatic lipid content and protects from diet-induced ectopic lipid accumulation in our *Slc16a13* knockout mouse model (Figs. 7 and 8). These results are consistent with previous studies that have demonstrated that elevation of liver-targeted mitochondrial uncoupling can reduce HFD-induced hepatic steatosis and hepatic insulin resistance^50,51^. In our model, hepatic Slc16a13 seems to be causative in this protection, as this transporter is highly expressed in the liver, but only marginally expressed in other metabolically active tissues, such as skeletal muscle (Fig. 1a). Furthermore, adipose and muscle insulin sensitivity is unaltered between *Slc16a13* knockout and wild-type mice, as determined by 2-deoxyglucose uptake during HE clamp (Fig. 6h). Most importantly, the proposed liver-specific effect is supported by the difference between the genotypes in mitochondrial respiration of primary hepatocytes and liver tissue (Fig. 8a, b), liver transcriptomics (Fig. 9b), and protein expression analyses (Fig. 9a). Opposite to these changes in liver metabolism, we could not detect altered mitochondrial function in skeletal muscle of knockout mice, indicated by transcriptomic data and respiratory protein expression (Supplementary Fig. 6). This may also explain the trend seen in whole-body energy expenditure whereby high oxygen consumption in the liver, which accounts for up to 50% of whole-body energy expenditure in mice^52^, but not skeletal muscle, may have a non-significant impact on total energy expenditure (Fig. 5c), which is consistent with prior observations using liver-specific mitochondrial uncoupling agents^50,51^. Although we assert altered hepatic β-oxidation to be causative for metabolic changes seen in the *Slc16a13* knockout mice, the respiratory exchange ratio addressed by indirect calorimetry is unchanged between the genotypes (Fig. 5f). However, as assessed for whole-body energy expenditure, also fatty acid oxidation may not be upregulated on a whole-body level if our proposed mechanism is liver-specific. Finally, the overall mild phenotype of *Slc16a13* knockout mice may also be at least partially explained by the observed upregulation of various Slc16 genes (Supplementary Fig. 9), maintaining hepatic lactate level above a critical threshold that may affect hepatic lipid and glucose metabolism more dramatically. Therefore, also biomarkers of liver and kidney function may not be altered in the *Slc16a13* knockout mouse model (Supplementary Fig. 11).

Our whole-body knockout mouse model cannot distinguish between Slc16a13 action in different organs and its organ-specific phenotypical consequences. In this study, we did not focus on the role of Slc16a13 in organs other than the liver. However, in obese mice, our data do not point to any impact of Slc16a13 deficiency on adipose tissue and skeletal muscle. In contrast to the liver of obese mice, adipose tissue and skeletal muscle did not show differences in lipid accumulation, as addressed by adipose tissue weights (Supplementary Table 3) and absolute triglyceride quantification in quadriceps muscle (Supplementary Fig. 6c). Moreover, insulin sensitivity is unaltered in adipose tissue and skeletal muscle (Fig. 6h), and finally, oxidative phosphorylation is altered in the liver, but not in skeletal muscle (Supplementary Fig. 6a, b), which accounts for ~10% of whole-body energy expenditure in mice^52^. Importantly, *Slc16a13* is only marginally expressed in adipose tissue and skeletal muscle (Fig. 1a and Supplementary Fig. 1a), and not differentially expressed comparing lean and obese mice (Supplementary Fig. 1c, d), speculating that the impact of Slc16a13 deficiency on these tissues may be low compared to tissues of high *Slc16a13* abundance and/or dysregulation in obese mice, as seen in the liver. Although our data clearly show an effect in the liver, we cannot completely rule out the possibility that deletion of *Slc16a13* in the kidney contributes to the effects on metabolism. Contrary to mice, human *SLC16A13* shows its highest expression in the liver, and kidney SLC16A13 may not be crucial in human metabolic disease. Additionally, we and others^3^ only investigated mRNA expression. Further investigations on SLC16A13 protein levels could help uncover the physiological function of the transporter in different tissues more clearly.

Our results are similar in some aspects to published data on *Slc16a1* knockout mice^32,40^. Although homozygous *Slc16a1* knockout mice are not viable, heterozygous knockout mice are protected from diet-induced obesity, insulin resistance, and hepatic steatosis^40^. Reduced weight gain was associated with decreased hepatic fat accumulation and proposed to be mediated by lower liver lactate metabolism and subsequent AMPK activation^32^. There are several advantages of SLC16A13/MCT13 over MCT1 in terms of a pharmacological target for the treatment of metabolic disease: First, homozygous deletion in mice leads to viable offspring, which is not the case in *Slc16a1* knockout mice. Second, *Slc16a1* is ubiquitously expressed and thus, MCT1 inhibition could have broader physiological side effects. Finally, and most importantly, GWAS identified *SLC16A13* as novel loci for T2D in one large cohort^17^.

Regarding the improved metabolic phenotype associated with loss of Slc16a13 function in our knockout mouse model, we propose the described human *SLC16A13* polymorphism linked to T2D to reflect a gain-of-function mutation. Interestingly, this may be opposite to previously reported *SLC16A11* polymorphisms where loss-of-function mutations leading to T2D are postulated^6,16^. The *SLC16A11* T2D risk variants have been shown to impact the expression of *SLC16A11* but not of *SLC16A13*^6^. Our work proposes *SLC16A13* gain-of-function genetic variants to increase T2D risk and raises the question about a potential causative role for SLC16A13 other than SLC16A11, highlighting the need of additional studies to better understand the distinct mechanism through which these SLC16 transporters affect the development of T2D. Finally, our study demonstrates that targeting SLC16A13 represents a promising therapeutic strategy for the treatment of fatty liver and insulin resistance at the same time.

## Methods

### Animals

*Slc16a13* heterozygous mice on C57BL/6N genetic background were generated by Taconic Biosciences using CRISPR/Cas9 technology. Mice were bred to homozygosity in the animal facility at the Experimental Centre of the Medical Faculty, TU Dresden. Animals were housed under standard conditions with a room temperature of 22 °C, humidity of 50–60%, 12 h light-dark cycle, and free access to water and food. *Slc16a13* knockout and littermate wild-type males were fed with normal-chow diet (NCD, 15% calories from fat, sniff, Soest) or high-fat diet (HFD, 60% calories from fat, sniff, Soest) at an age of 5 weeks to 20 weeks. In vivo experiments were performed after an overnight fast at the indicated ages and if not stated otherwise, mice were killed after an overnight fast at 20 weeks of age. If not stated otherwise, parameters were investigated at 15 weeks of HFD-feeding and one data set represents one experiment on a single cohort of animals, whereat multiple cohorts have been analyzed. All procedures were approved by the Landesdirektion Sachsen (TVV 59/2015).

### Metabolic monitoring

Fat and lean mass were determined by ^1^H nuclear magnetic resonance using the minispec LF50 body composition analyzer (Bruker). The TSE labmaster caging system (TSE systems) was used to analyze O_2_ consumption (VO_2_) and CO_2_ production (VCO_2_) by indirect calorimetry, energy expenditure as calculated by (VO_2_ × 15.818) + (VCO_2_ × 5.176), locomotor activity as well as food intake.

### Oral glucose tolerance test

40% glucose solution was administered by gavage (2 mg glucose per gram body weight) and blood glucose was determined prior and 15, 30, 60, and 120 min after application. Blood was taken from the tail by massage technique.

### Hyperinsulinemic euglycemic clamp

Insulin sensitivity of the animals was investigated using the hyperinsulinemic–euglycemic clamp as described previously^53^. Intravenous lines were placed into the right vena jugularis externa. One week after surgery, mice were continuously infused with 0.05 μCi/min [3-^3^H]glucose (Perkin Elmer) over 2 h to assess basal glucose turnover. Hyperinsulinemic euglycemic clamps were conducted over 140 min with a primed insulin bolus of 21 mU/kg over 3 min, followed by continuous infusion of 3 mU/(kg-min) insulin and a variable infusion of 20% dextrose to maintain euglycemia (120 mg/dl). 0.1 μCi/min [3-^3^H]glucose was continuously infused to determine whole-body glucose uptake and insulin suppression of endogenous glucose production after the basal period. 10 μCi bolus of 2-deoxy-d-[1-^14^C]glucose (Perkin Elmer) was injected after 85 min for the determination of the insulin-stimulated tissue glucose uptake into individual insulin-sensitive organs, i.e., skeletal muscle and adipose tissue.

### De novo lipogenesis

Hepatic de novo lipogenesis was assessed as reported previously^54^. Mice were injected i.p. with 23.4 µl deuterated saline (0.9% saline dissolved in 99% ^2^H_2_O-enriched water, Sigma-Aldrich) per gram body weight. At this time, drinking water was replaced with 6% ^2^H_2_O, 1% sucrose. After three nights, mice were fasted for 6 h, killed and liver tissue and whole blood were collected. Triglycerides were extracted from the frozen liver by a chloroform-methanol extraction. Samples in chloroform were purified by silica gel thin-layer chromatography. Triglyceride-fatty acids were analyzed by GC–MS as fatty acid methyl esters following derivatization with methanolic boron trifluoride. The plasma ^2^H_2_O pool was assessed by exchange of hydrogens from plasma to acetone in the presence of sodium hydroxide; acetone deuterium enrichment was analyzed by GC–MS.

### VLDL export

Hepatic VLDL-triglyceride secretion was analyzed as described before^55^. Briefly, blood was taken from the tail prior to and 1, 2, 3, and 4 h after i.p. injection of 0.001 g Poloxamer-407 (Sigma-Aldrich, dissolved in 0.9% saline) per gram body weight. Plasma triglycerides were determined by an enzymatic method.

### Insulin stimulation

Mice were injected i.p. with 7.5 mU insulin per gram body weight or 0.9% saline as control. Liver tissue was sampled 20 min after injection.

### Generation of HEK-*Slc16a13* cells

The cDNA encoding mouse *Slc16a13* was cloned using a PCR-based approach. The primer pair 5′-TGT-TCC-TTT-CGG-AAT-GGT-GCG-3′ and 5′-CGG-TGG-AGT-TTA-GTT-CTC-TCC-C-3′ for HEK-*Slc16a13* or 5′-TCA-CTT-GTC-GTC-ATC-GTC-TTT-GTA-GTC-GTT-CTC-TCC-CAG-CCC-CTC-C-3′ for HEK-*Slc16a13-FLAG* was used to amplify *Slc16a13* from mouse liver cDNA. Amplified fragments were cloned into pcDNA3.1(+) expression plasmid. HEK293 cells were stably transfected with mouse Slc16a13 expression plasmid (HEK-*Slc16a13*), Slc16a13-FLAG expression plasmid (HEK-*Slc16a13-FLAG*) or the empty vector (HEK-control) using Effectene transfection reagent (Qiagen) and selected using geneticin (800 µg/ml). Overexpression of *Slc16a13* in the HEK-*Slc16a13* and HEK-*Slc16a13-FLAG* cell line compared to HEK-control cells was determined by qRT-PCR.

### Immunofluorescence staining

HEK-*Slc16a13-FLAG* or HEK-control cells were seeded onto poly-D-lysine coated cover glasses, fixed in 4% PFA, blocked with 10% goat normal serum in PBS, and incubated with the following antibodies and dyes: FLAG (ThermoFisher Scientific, PA1-984B, 1:500), Golgin-97 (ThermoFisher Scientific, A-21270, 1:100), Calnexin (Novus Biologicals, NB300-518, 1:100), goat anti-rabbit Alexa Fluor 488 secondary antibody (ThermoFisher Scientific, A-11008, 1:500), goat anti-mouse Alexa Fluor 594 (ThermoFisher Scientific, R37121, 1:500), Alexa Fluor 594 wheat germ agglutinin (ThermoFisher Scientific, W11262, 1:100), MitoTracker CMXRos (ThermoFisher Scientific, M7512, 100 nM) and Hoechst 33342 (Invitrogen, 1:2000). Microscopy has been performed at the Leica TCS SP5 confocal laser scanning microscope (Leica Microsystems).

### l-lactate uptake

Uptake experiments were performed as previously described^56^. In brief, HEK-*Slc16a13* and HEK-control cells were seeded onto poly-d-lysine coated 12-well plates. After three days, cells are washed twice with pre-warmed uptake buffer (120 mM NaCl, 5.4 mM KCl, 0.8 mM MgSO_4_, 5 mM glucose, 1.8 mM CaCl_2_, 25 mM HEPES, pH 7.3) and incubated in uptake buffer for 2 min at 37 °C with the indicated concentrations of unlabeled l-lactic acid spiked with l-[14 C(U)]-Lactic Acid (Perkin Elmer). Cells were washed three times with ice-cold uptake buffer before lysing the cells with 0.2% SDS. ^14^C l‑lactic acid in the whole-cell lysate was detected by liquid scintillation counting. Uptake experiments were performed three times in triplicates. All transport rates were normalized to protein content. The transport of l-lactate in HEK-control cells was set to 100% to calculate l-lactate uptake of HEK-*Slc16a13* cells (%). Net l-lactate uptake was calculated as the difference in transport between HEK-*Slc16a13* cells and HEK-control cells. *K*_m_ value was assessed using nonlinear regression curve fit (GraphPad Prism 7).

### Isolation of mouse primary hepatocytes

Isolation of hepatocytes was performed as previously described^57^. Hepatocytes were isolated from NCD-fed female mice at a mean age of 20 weeks. Mice were anesthetized by i.p. injection of ketamine-xylazine. Livers were perfused via the vena cava with pre-warmed perfusion buffer (Earle’s Balanced Salt Solution w/o CaCl_2_/MgCl_2_) supplemented with 0.5 mM EGTA, followed by perfusion with pre-warmed digestion buffer (5000 U collagenase in phenol red-free Hank’s Salt Solution). The liver was resected and mashed in Dulbecco’s modified Eagle’s medium (DMEM) w/o pyruvate containing 25 mM glucose, 10% fetal calf serum (FCS), and 1% penicillin/streptomycin. The cell suspension was filtered through a 100 µM mesh and hepatocytes were purified by density gradient centrifugation using Percoll solution (GE Healthcare).

### Cell culture

Primary hepatocytes and HEK293 cells were cultured in DMEM containing 25 mM glucose, 10% FCS, and 1% penicillin/streptomycin. For the culture of HEK cell lines, geneticin (800 µg/ml) was added to the medium. Cells were cultured in an incubator at 37 °C and 5% CO_2_. Cell culture media, supplements, and buffers are purchased from Gibco unless otherwise stated.

### Human liver samples

Liver biopsies from patients undergoing partial hepatectomy were taken from a segment without pathological findings as reported previously^57–59^. The tissue was snap-frozen immediately and processed for mRNA expression analysis. Medical history, physical parameters like age, BMI, fat mass, and waist circumference as well as clinical parameters including fasting blood glucose and insulin levels to calculate the HOMA-IR have been recorded for all patients. Histopathology was carried out by evaluating the NAFLD-activity score (NAS), which uses the criteria steatosis, inflammation, hepatocyte ballooning, and fibrosis. Subjects were then characterized according to steatosis severity: steatosis score = 0 (0–<5% steatosis); steatosis score = 1 (5–33% steatosis); and steatosis score ≥ 2 (>33% steatosis)^60^. Excessive ethanol consumption was considered to be >20 g/day for women and >40 g/day for men. Metabolic syndrome and type 2 diabetes were diagnosed according to accepted criteria. Clinical chemistry was measured in fasting blood samples in certified laboratories. Whole-body insulin resistance was estimated by the homeostasis model assessment of insulin resistance^61^. Characteristics of the 45 included patients from the cross-sectional INSIGHT study are shown in Supplementary Table 1. Informed consent was obtained from each patient included in the study, and the study protocol conforms to the ethical guidelines of the 1975 Declaration of Helsinki as reflected in approval by the ethics committee of the Charité-Universitätsmedizin Berlin (ethics approval number: EA2/135/08, German Clinical Trials Register: DRKS00005450).

### qRT-PCR

Total RNA was extracted from frozen liver tissue homogenates using Trizol reagent (Sigma-Aldrich). cDNA was synthesized using the RevertAid First Strand cDNA Synthesis Kit and the qRT-PCR reaction was performed using SYBR Green (both ThermoFisher Scientific) and the CFX384 Touch Real-Time PCR Detection System (Bio-Rad). Procedures were performed according to the manufacturer’s instructions. Ct-values were normalized to the housekeeper *HPRT*. Primer sequences are shown in Supplementary Table 4.

### H&E- and Oil-Red-O staining

Liver tissue was fixed in 4% PFA, embedded in paraffin, and cut into 3 µM sections. Liver sections were deparaffinized, rehydrated, and stained with hematoxylin–eosin (H&E) for histological evaluation of liver morphology. For visualization of liver lipids, the liver tissue was fixed, dehydrated in 20% sucrose solution, and embedded in TissueTek (Sakura Finetek) for cryostat sectioning. 15 µM-thick liver sections were stained with freshly prepared Oil-Red-O solution (Sigma-Aldrich, 3 mg/ml). Microscopy has been performed at the Axio Observer Z.1 widefield microscope using the Zen 2.3 software (Carl Zeiss microscopy).

### Triglyceride extraction

Liver and muscle tissue were homogenized in 0.9% saline. Triglycerides were isolated using a 2:1 chloroform-methanol solution. Evaporated lipid phase was dissolved in isopropanol and quantified using an enzymatic assay.

### Diacylglycerol and ceramide extraction

Hepatic diacylglycerols and ceramides were analyzed using liquid chromatography and tandem mass spectrometry as described before^62^.

### PKCɛ translocation assay

Membrane-associated and cytosolic fractions were prepared as previously described^23^. Following western blotting, the ratio of membrane PKCε intensity (normalized to Na-K ATPase) to cytosolic PKCε intensity (normalized to GAPDH) was calculated.

### Palmitate stimulation of mouse primary hepatocytes

Hepatocytes were seeded in collagen-coated 6-well plates (700,000 cells/well). After incubation for 5 h, DMEM was changed and cells were incubated overnight. The next day, the cell culture medium was replaced by DMEM without FCS and supplemented with 5% lipid-depleted serum and 0.5% fatty acid-free BSA or 500 µM fatty acid-free BSA-coupled palmitate (~6:1 molar ratio). After incubation for 18 h, cells were harvested for western blot analysis of AMPK and ACC phosphorylation.

### Glucose production of mouse primary hepatocytes

In vitro gluconeogenesis was analyzed as previously described^63^ with minor changes. In short, hepatocytes were seeded in collagen-coated 12-well plates (300,000 cells/well) in DMEM high-glucose medium (25 mM glucose). After incubation for 5 h, cells were washed with PBS and incubated for 16 h in low-glucose DMEM (5.55 mM glucose) supplemented with 0.5% fatty acid-free BSA or 500 µM fatty acid-free BSA-coupled palmitate (~6:1 molar ratio). The next morning, cells were washed twice with PBS and cell culture media was replaced with 0.5 ml glucose production medium (DMEM base medium supplemented with 20 mM sodium lactate, 2 mM sodium pyruvate, and 10 mM HEPES, pH 7.4) in the presence of 500 µM BSA-coupled palmitate or 0.5% BSA control, and 100 nM glucagon or vehicle control. After incubation for 8 h, the cell culture medium was collected for analysis of glucose, and cells were harvested for protein normalization.

### Oxygen consumption of mouse primary hepatocytes

Mouse primary hepatocytes were seeded in collagen-coated XF96 cell culture microplates (10,000 cells/well) and incubated overnight. On the day of the experiment, the cell culture medium was replaced by Seahorse assay medium (DMEM base medium supplemented with 31.6 mM NaCl, 1 mM sodium pyruvate, 2 mM l-glutamine, 10 mM glucose, pH 7.4). The Seahorse XF Cell Mito Stress Test was performed on the Seahorse XF96 Extracellular Flux Analyzer (Agilent Technologies) according to the manufacturer’s instructions using 3 µM oligomycin, 0.5 µM FCCP, 5 µM rotenone, and 5 µM antimycin A (Seahorse Bioscience). The oxygen consumption rate was normalized to protein content. The following states have been analyzed: basal respiration = OCR at baseline conditions, ATP production = OCR calculated as oligomycin-inhibited OCR subtracted from basal respiration, maximal respiration = OCR after FCCP-mediated uncoupling, spare respiratory capacity = OCR calculated as basal OCR subtracted from maximal OCR, and non-mitochondrial respiration = rotenone/ antimycin A-inhibited OCR.

### Oxygen flux of mouse liver tissue

2 mg fresh liver was used for high-resolution respirometry with the Oroboros O2k oxygraph (Oroboros Instruments). SUIT protocol was performed according to the manufacturer’s instructions using 2 mM malate, 1 mM octanoylcarnitine, 2.5 mM ADP, 10 mM glutamate, 5 mM succinate, 0.25 µM steps of FCCP titration, and 5 µM antimycin A. Oxygen flux was normalized to tissue mass. The respiration states were defined according to the following substrate injections: LEAK = proton leak, ion leak and slip compensatory state (O_2_ flux after malate-octanoylcarnitine injection), FAO = fatty acid oxidation capacity (O_2_ flux after ADP injection), CI_OXPHOS_ = complex I-related oxidative phosphorylation capacity (O_2_ flux after glutamate injection), CI + II_OXPHOS_ = complex I and III-related oxidative phosphorylation capacity (O_2_ flux after succinate injection), ETS = electron transfer system capacity (maximum O_2_ flux after FCCP titration), and ROX = residual oxygen consumption (minimum O_2_ flux after antimycin A injection).

### Biochemical assays

Blood glucose levels were determined using the Accu-Chek Aviva Plus test system (Roche Diagnostics). Plasma insulin levels were analyzed using the Ultra Sensitive Mouse Insulin ELISA (Crystal Chem). Liver and plasma triglyceride levels were measured using the LabAssay Triglyceride kit (FUJIFILM Wako Pure Chemical Corp.). Plasma-free fatty acids were quantified using the NEFA-HR(2) assay (FUJIFILM Wako Pure Chemical Corp.). Plasma l-lactic acid concentrations were determined using the coloriometric l-lactate assay kit (Abcam). Plasma ALT, AST, creatinine, urea, and albumin as well as glucose in cell culture supernatant were measured using the cobas 8000 modular analyzer (Roche Diagnostics). All measurements were performed according to the manufacturer’s instructions.

### Western blot

Tissue was lysed in RIPA buffer (50 mM Tris-HCl, pH 7.4, 150 mM NaCl, 1% Nonidet P-40, 0.5% sodium deoxycholate, 0.1% SDS) containing protease and phosphatase inhibitors (Life Technologies). 10–50 µg protein lysate was separated on 10% SDS-PAGE gels. For the Slc16a13 western blot, the plasma membrane was extracted using the Abcam kit ab65400 according to the manufacturer’s instructions, and 90 µg membrane protein was loaded on the SDS-PAGE gel. Protein gels were transferred to methanol-activated PVDF membranes using the wet electrophoretic transfer system (Bio-Rad). The membrane was blocked with 5% BSA or milk powder in TBS/0.1% Tween, primary antibodies were incubated at 4 °C overnight and secondary antibodies for one hour at room temperature. Peroxidase-conjugated secondary antibodies and Western Bright Chemiluminescence Substrate Sirius (Biozym) were used for protein visualization using the Fusion FX (Vilber Lourmat) and Fusion FX7 Advance imaging software. Densitometric analysis was carried out using ImageJ software (NIH). The following antibodies have been used: SLC16A13 (ThermoFisher Scientific, PA5-39249, 1:500), Vinculin (Abcam, ab129002, 1:10,000), GAPDH (Abcam, ab8245, 1:20,000), Na-K ATPase (Abcam, ab7671; 1:1000), PKCε (BD Biosciences, 610086, 1:1000), Acetyl Coenzyme A Carboxylase (Abcam, ab45174, 1:2000), Acetyl Coenzyme A Carboxylase (phospho S79) (Abcam, ab68191, 1:5000), total OXPHOS Rodent WB Antibody Cocktail (Abcam, ab110413, 1:1000), AMPK alpha 1 + AMPK alpha 2 antibody (Abcam, ab207442, 1:1000), AMPK alpha 1 (phospho T183) + AMPK alpha 2 (phospho T172) (Abcam, ab133448, 1:1000), Akt (pan) (Cell Signaling, 4691, 1:1000), Phospho-Akt (Ser473) (Cell Signaling, 4060, 1:1000), goat anti-rabbit IgG, HRP conjugated (Millipore, 401353, 1:10,000), goat anti-mouse IgG, HRP conjugated (Millipore, 401253, 1:10,000).

### Sequencing

For next-generation sequencing, RNA quality and concentration were measured using a High Sensitivity Total RNA kit on a fragment analyzer (Agilent Technologies, Santa Clara, CA). RNA quality was high with RNA Integrity (RIN) values greater than 8.5 for all samples. For the library preparation, 50 ng of total RNA was used with the NEBNext Ultra II Directional RNA Library Prep Kit for Illumina (E7760L) using the NEBNext Poly(A) mRNA Magnetic Isolation Module from (NEB #E7490) and the dual index plate (NEB # E6440L) from New England BioLabs Inc. We obtained 350 bp fragments including adapters in average size. For multiplexing, 11 individual libraries were normalized and pooled. The pooled libraries were clustered using the Illumina cBot Instrument (TruSeq SR Cluster Kit v3—cBot—HS (cat# GD-401-3001; Illumina Inc.)). Single-end reads were sequenced at a length of 85 bp with 8 bases dual index reads on an Illumina HiSeq4000 instrument (TruSeq SBS Kit HS- v3, 50-cycle, cat# FC-401-3002; Illumina Inc.). All samples reached a sequencing depth of 30–35 million reads per sample.

The limma package^64^ in the R programming environment was used to convert raw counts to log2-counts per million, normalized to account for the systematic mean-variance relationships often present in microarray and RNA-Seq data. A Wilcoxon Rank Sum-based gene set enrichment analysis was performed using the algorithm provided in the gage package^65^, which was designed for use on microarray and RNA-Seq data. All *p*-values were corrected for False Discovery Rate.

### Metabolomics

Metabolomics analysis on mouse liver extracts and serum was performed by the U.C. Davis West Coast Metabolomics Center as previously described^66^. In brief, liver tissue (4 mg) was homogenized in extraction solution (acetonitrile:isopropanol:water, 3:3:2), vortexed for 45 s, and incubated for 5 min at 4 °C. Following centrifugation at 14,000×*g* for 2 min, aliquots were dried via evaporation overnight. The dried aliquot was resuspended in 50% acetonitrile (degassed) and centrifuged at 14,000×*g* for 2 min. The supernatant was evaporated again to dryness. Internal standards (C8-C30 fatty acid methyl esters) were added and the sample was derivatized by methoxyamine hydrochloride in pyridine and subsequently by *N*-methyl-*N*-trimethylsilyl trifluoro-acetamide for trimethylsilylation of acidic protons. Data were acquired using the method as described^67^, which is briefly summarized in Mitchell et al.^68^. Data were presented as a ratio of the metabolite to the total metabolites returned. Metabolite profiles were analyzed using MetaboAnalyst versions 3.0 and 4.0^69,70^, utilizing univariate and multivariate built-in analytical methods from modules of this web-based platform, as specified. Differential expression analysis was performed with the limma package^64^ in the R programming environment and an unpaired two-tailed *t*-test with Welch’s correction of individual metabolites was performed by considering the genotype.

### Statistics and reproducibility

Data analysis was performed using GraphPad Prism 7 software. A two-tailed unpaired Student’s *t*-test was used to compare the mean of two groups. One-tailed unpaired Student’s *t*-test was used for hypothesis testing of higher or lower than the control mean. One-way ANOVA followed by Dunnett’s multiple comparison test was used to compare means of more than three groups to the control group. Two-way ANOVA followed by Sidak’s multiple comparison test was used to compare two groups with repeated measurements. Statistical significance is stated as follows: *p < 0.05, **p < 0.01, ***p < 0.001. Different cohorts of *Slc16a13* knockout and wild-type littermates were included in this study in order to conform animal welfare and secure the reproducibility of the metabolic phenotype.

### Reporting summary

Further information on research design is available in the Nature Research Reporting Summary linked to this article.

## Supplementary information

Supplementary Information

Description of Additional Supplementary Files

Supplementary Data 1

Reporting Summary

## Data Availability

Source data are available in Supplementary Data 1. RNA-Seq data have been deposited to the Gene Expression Omnibus (GEO) and can be accessed via the accession number GSE152937. The metabolomics data set has been deposited to the Metabolomics Workbench (study ID ST001643, project 10.21228/M8V702). Other data that support the findings of the study are available from the corresponding author on reasonable request.
