# Publisher Correction: Deletion of the diabetes candidate gene *Slc16a13* in mice attenuates diet-induced ectopic lipid accumulation and insulin resistance

**DOI:** 10.1038/s42003-021-02425-2

**Published:** 2021-07-14

**Authors:** Tina Schumann, Jörg König, Christian von Loeffelholz, Daniel F. Vatner, Dongyan Zhang, Rachel J. Perry, Michel Bernier, Jason Chami, Christine Henke, Anica Kurzbach, Nermeen N. El-Agroudy, Diana M. Willmes, Dominik Pesta, Rafael de Cabo, John F. O´Sullivan, Eric Simon, Gerald I. Shulman, Bradford S. Hamilton, Andreas L. Birkenfeld

**Affiliations:** 1 Section of Metabolic and Vascular Medicine, Medical Clinic III, Dresden University School of Medicine, Technische Universität Dresden, Dresden, Germany; 2 Paul Langerhans Institute Dresden of the Helmholtz Center Munich at University Hospital and Faculty of Medicine, Technische Universität Dresden, Dresden, Germany; 3 German Center for Diabetes Research (DZD), Neuherberg, Germany; 4 Clinical Pharmacology and Clinical Toxicology, Institute of Experimental and Clinical Pharmacology and Toxicology, Friedrich-Alexander-Universität Erlangen-Nürnberg, Erlangen, Germany; 5 Department of Anaesthesiology and Intensive Care, Jena University Hospital, Jena, Germany; 6 Department of Internal Medicine, Yale School of Medicine, New Haven, CT USA; 7 Department of Cellular and Molecular Physiology, Yale School of Medicine, New Haven, CT USA; 8 Experimental Gerontology Section, Translational Gerontology Branch, National Institute on Aging, National Institutes of Health, Baltimore, MD USA; 9 Heart Research Institute, Newtown, NSW Australia; 10 Institute of Aerospace Medicine, German Aerospace Center (DLR), Cologne, Germany; 11 Centre for Endocrinology, Diabetes and Preventive Medicine (CEDP), University Hospital Cologne, Cologne, Germany; 12 Institute for Clinical Diabetology, German Diabetes Center, Leibniz Center for Diabetes Research at Heinrich-Heine University Düsseldorf, Düsseldorf, Germany; 13 Charles Perkins Centre, The University of Sydney, Camperdown, NSW Australia; 14 Department of Cardiology, Royal Prince Alfred Hospital, Camperdown, NSW Australia; 15 Computational Biology, Boehringer-Ingelheim Pharma GmbH & Co. KG, Biberach an der Riss, Germany; 16 CardioMetabolic Diseases Research, Boehringer-Ingelheim Pharma GmbH & Co. KG, Biberach an der Riss, Germany; 17 King’s College London, Department of Diabetes, School of Life Course Science, London, UK; 18 Institute for Diabetes Research and Metabolic Diseases of the Helmholtz Centre Munich at the University of Tübingen, Tübingen, Germany; 19 Department of Endocrinology, Diabetology and Nephrology, University Hospital of Tübingen, Tübingen, Germany

**Keywords:** Type 2 diabetes, Fat metabolism, Animal disease models, Non-alcoholic fatty liver disease

Correction to: *Communications Biology* 10.1038/s42003-021-02279-8, published online 1 July 2021.

The original published version of the Article contained an error in the abstract whereby the words “loss of” were inadvertently omitted from the following sentence: “We show that loss of Slc16a13 increases mitochondrial respiration in the liver, leading to reduced hepatic lipid accumulation and increased hepatic insulin sensitivity in high-fat diet fed *Slc16a13* knockout mice.” The error has been corrected in the HTML and PDF versions of the Article.

