# Supplementary material for: Deletion of the diabetes candidate gene Slc16a13 in mice attenuates diet-induced ectopic lipid accumulation and insulin resistance
Source: Commun Biol. 2021 Jul 1;4:826. doi: 10.1038/s42003-021-02279-8 (PMC8249653; doi:10.1038/s42003-021-02279-8)
Supplement: Supplementary file 1 — Supplementary Information [file 42003_2021_2279_MOESM1_ESM.pdf]

Supplementary Information

Deletion of the diabetes candidate gene *Slc16a13* in mice attenuates diet-induced ectopic lipid accumulation and insulin resistance

Tina Schumann[1,2,3], Jörg König[4], Christian von Loeffelholz[5], Daniel F. Vatner[6], Dongyan Zhang[6], Rachel J. Perry[6,7], Michel Bernier[8], Jason Chami[9], Christine Henke[1,2,3], Anica Kurzbach[1,2,3], Nermeen N. El-Agroudy[1,2,3], Diana M. Willmes[1,2,3], Dominik Pesta[10,11,12,3], Rafael de Cabo[8], John F. O´Sullivan[9,13,14], Eric Simon[15], Gerald I. Shulman[6,7], Bradford S. Hamilton[16] & Andreas L. Birkenfeld[1,3,17,18,19*]

[1] Section of Metabolic and Vascular Medicine, Medical Clinic III, Dresden University School of Medicine, Technische Universität Dresden, Germany
[2] Paul Langerhans Institute Dresden of the Helmholtz Center Munich at University Hospital and Faculty of Medicine, Technische Universität Dresden, Dresden, Germany
[3] German Center for Diabetes Research (DZD), Neuherberg, Germany
[4] Clinical Pharmacology and Clinical Toxicology, Institute of Experimental and Clinical Pharmacology and Toxicology, Friedrich-Alexander-Universität Erlangen-Nürnberg, Erlangen, Germany
[5] Department of Anaesthesiology and Intensive Care, Jena University Hospital, Jena, Germany
[6] Department of Internal Medicine, Yale School of Medicine, New Haven, Connecticut, USA
[7] Department of Cellular and Molecular Physiology, Yale School of Medicine, New Haven, Connecticut, USA
[8] Experimental Gerontology Section, Translational Gerontology Branch, National Institute on Aging, National Institutes of Health, Baltimore, Maryland, USA
[9] Heart Research Institute, Newtown, New South Wales, Australia
[10] Institute of Aerospace Medicine, German Aerospace Center (DLR), Cologne, Germany
[11] Centre for Endocrinology, Diabetes and Preventive Medicine (CEDP), University Hospital Cologne, Cologne, Germany
[12] Institute for Clinical Diabetology, German Diabetes Center, Leibniz Center for Diabetes Research at Heinrich-Heine University Düsseldorf, Düsseldorf, Germany
[13] Charles Perkins Centre, The University of Sydney, Camperdown, New South Wales, Australia
[14] Department of Cardiology, Royal Prince Alfred Hospital, Camperdown, New South Wales, Australia
[15] Computational Biology, Boehringer-Ingelheim Pharma GmbH & Co. KG, Biberach an der Riss, Germany
[16] CardioMetabolic Diseases Research, Boehringer-Ingelheim Pharma GmbH & Co. KG, Biberach an der Riss, Germany
[17] King´s College London, Department of Diabetes, School of Life Course Science, London, UK
[18] Institute for Diabetes Research and Metabolic Diseases of the Helmholtz Centre Munich at the University of Tübingen, Tübingen, Germany
[19] Department of Endocrinology, Diabetology and Nephrology, University Hospital of Tübingen, Tübingen, Germany

* **Corresponding author:**
Prof. Dr. med. Andreas L. Birkenfeld
Section of Metabolic and Vascular Medicine, Medical Clinic III, Dresden University School of Medicine
Technische Universität Dresden
Fetscherstraße 74
01307 Dresden
Germany
Tel. +49 (0)351 458-3651
Fax: +49 (0)351 458-3652
E-mail: Andreas.Birkenfeld@uniklinikum-dresden.de

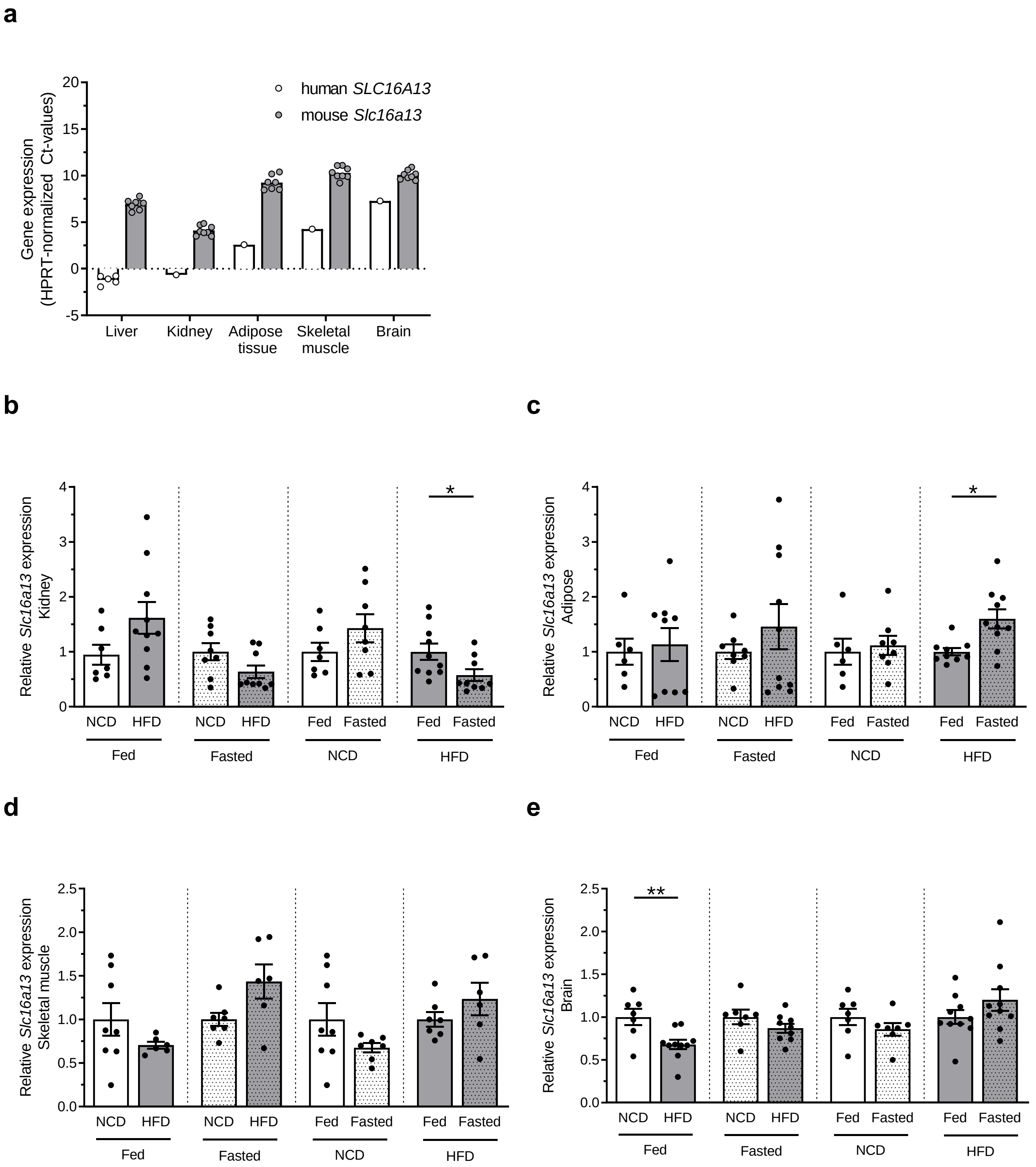

Supplementary Fig. 1 SLC16A13 expression in humans and mice. a HPRT-normalized Ct-values of human *SLC16A13* and mouse *Slc16a13* mRNA expression according to Fig. 1a. **b-e** Relative *Slc16a13* mRNA expression in kidney, white adipose tissue, skeletal muscle and brain (cortex) of C57BL/6J mice fed with NCD or HFD for 15 weeks. Animals are fed (n=6-10 per group) or fasted for 16 hours (n=6-9 per group). Bars represent means ± SEM. *p<0.05, **p<0.01 determined using two-tailed unpaired Student´s t-test.

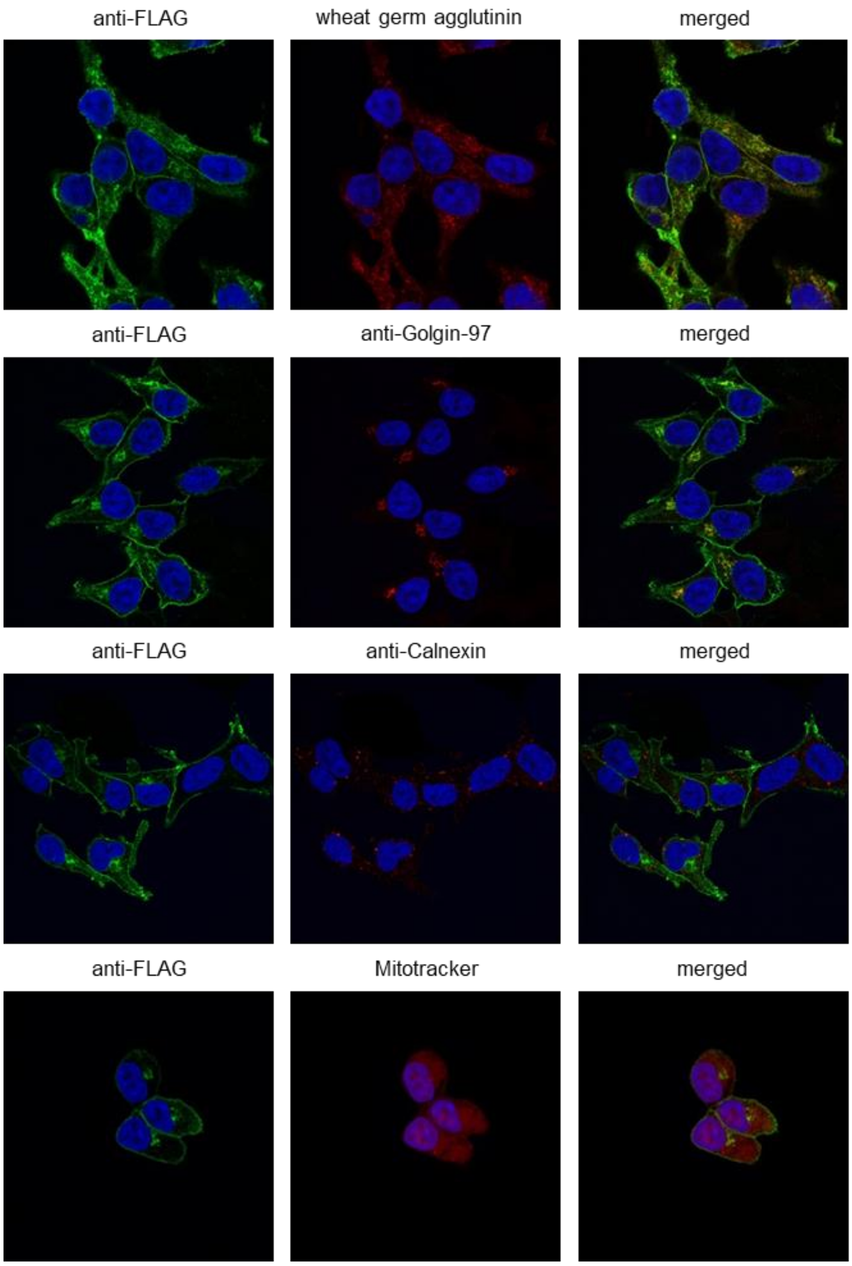

Supplementary Fig. 2 Mouse Slc16a13 cellular localization. Immunofluorescence co-staining of FLAG-tagged Slc16a13 with wheat germ agglutinin, Golgin-97, Calnexin or Mitotracker in HEK-m*Slc16a13-FLAG* cells. Representative stainings are shown.

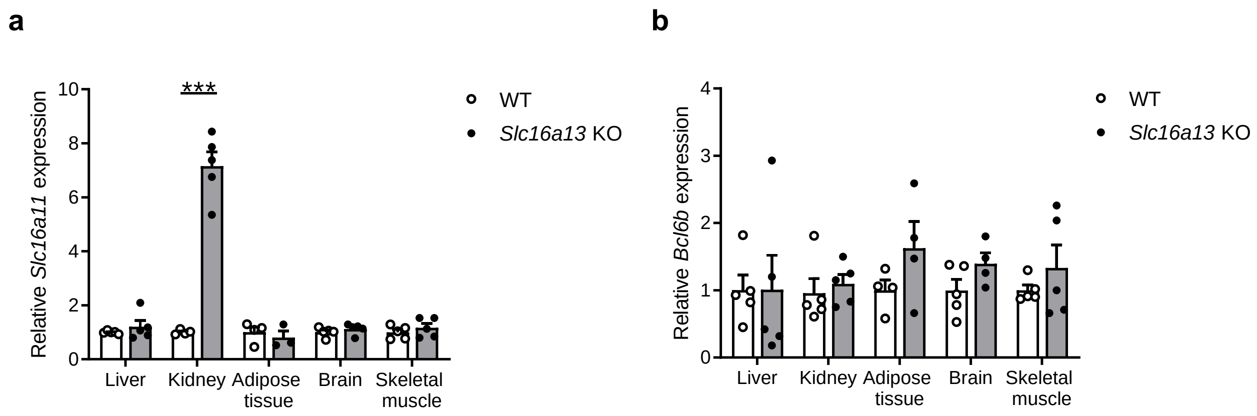

Supplementary Fig. 3 Gene expression of *Slc16a13* neighboring genes on mouse chromosome 11. Relative *Slc16a11* (**a**) and *Bcl6b* (**b**) mRNA expression in NCD-fed *Slc16a13* KO mice and WT controls (n=4-5 for each genotype and tissue). Bars represent means ± SEM. ***p<0.001 determined using two-tailed unpaired Student´s t-test.

a

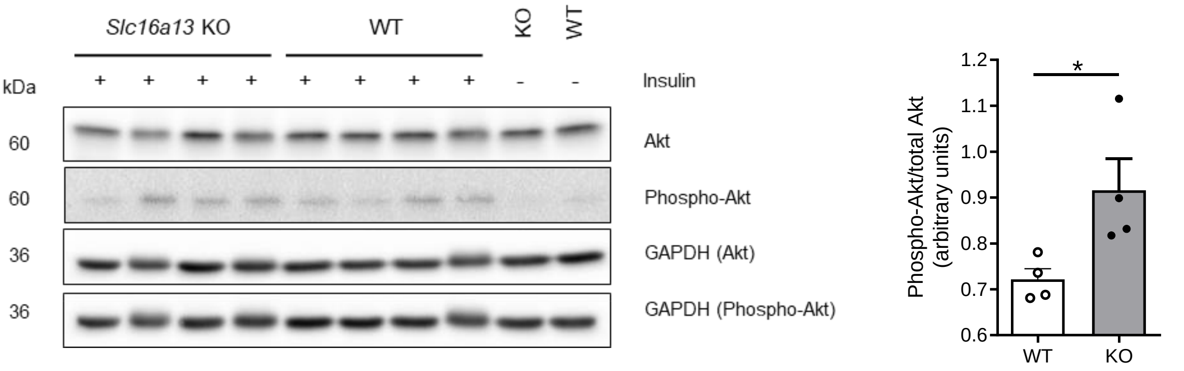

b

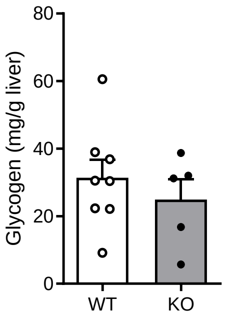

Supplementary Fig. 4 Hepatic glucose metabolism of *Slc16a13* knockout mice at 15 weeks of HFD. a Hepatic Akt phosphorylation as ratio of phospho-Akt/total Akt determined by Western Blot. *Slc16a13* KO (n=4) and WT (n=4) mice were sacrificed 20 minutes after insulin bolus. **b** Glycogen storage in livers of *Slc16a13* WT (n=8) and *Slc16a13* KO (n=5) mice. Bars represent means ± SEM.

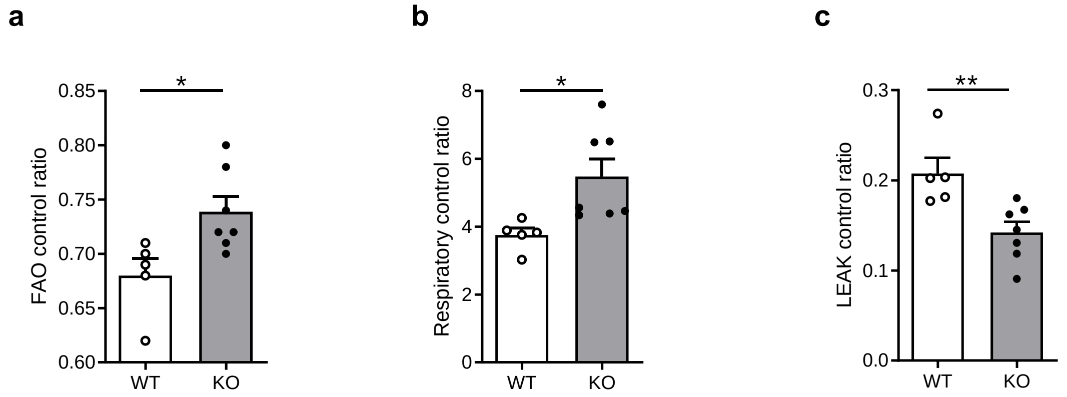

Supplementary Fig. 5 Respiratory control ratios in liver tissue of *Slc16a13* knockout mice at 15 weeks of HFD. Calculated ratios of oxygen flux during high-resolution respirometry performed in the Oroboros Oxygraph-2k (see Fig. 8b). **a** Fatty acid OXPHOS coupling control factor, calculated as (FAO-LEAK)/FAO using the fatty acid oxidation OXPHOS capacity and LEAK respiration. **b** Respiratory control ratio, calculated as $CI+II_{OXPHOS}$/LEAK using the $CI+II_{OXPHOS}$ and LEAK respiration. **c** LEAK control ratio, calculated as LEAK/ETS using the LEAK and ETS respiration. LEAK = proton leak, ion leak and slip compensatory state; FAO = fatty acid oxidation; $CI+II_{OXPHOS}$ = complex I and II-related oxidative phosphorylation capacity; ETS = electron transfer system; *Slc16a13* WT (n=5), *Slc16a13* KO (n=7). Bars represent means ± SEM. *p<0.05, **p<0.01 determined using two-tailed unpaired Student´s t-test.

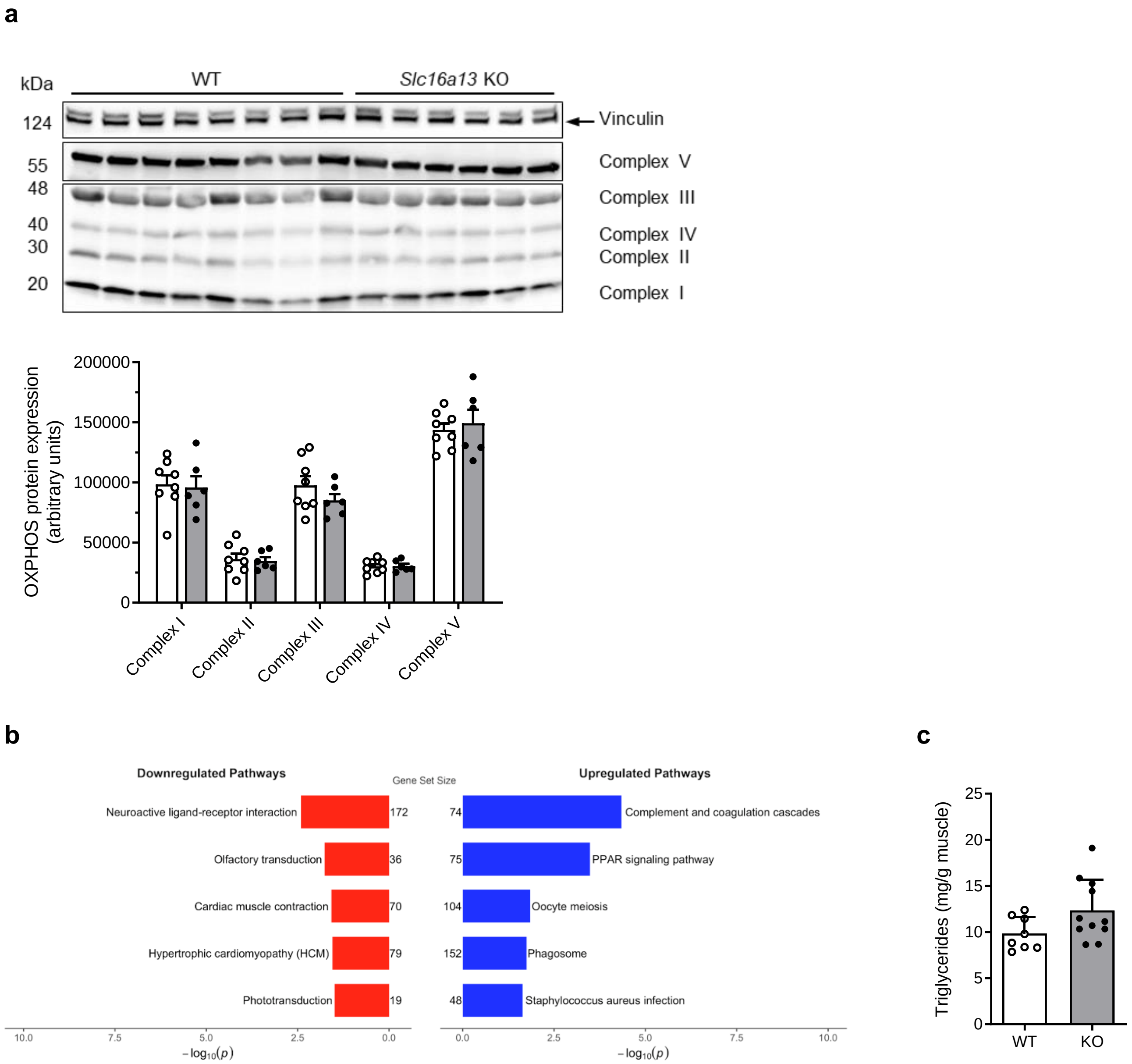

Supplementary Fig. 6 Molecular insight into unaltered mitochondrial respiration in skeletal muscle of *Slc16a13* knockout mice at 15 weeks of HFD. a Skeletal muscle protein expression of respiratory chain complexes I-V determined by Western Blot. **b** Gene set enrichment analysis of muscle transcriptomic data from WT and *Slc16a13* KO mice. **c** Triglycerides in skeletal muscle of WT and *Slc16a13* KO mice. *Slc16a13* WT (n=8-11), *Slc16a13* KO (n=6-11). **a, c** Bars represent means ± SEM.

a

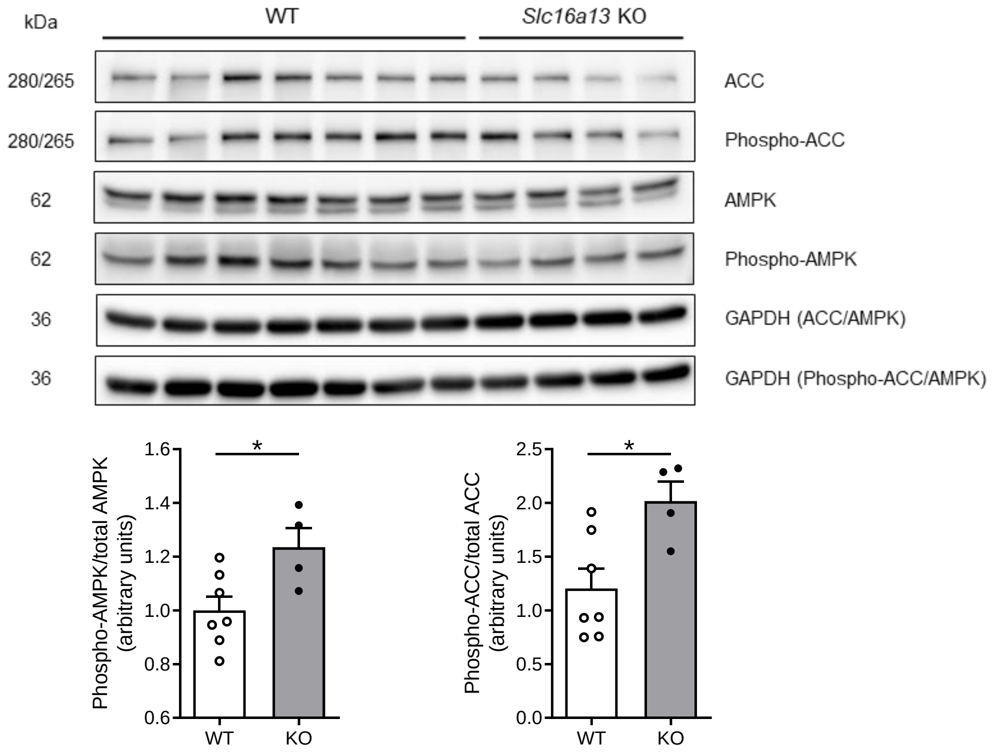

b

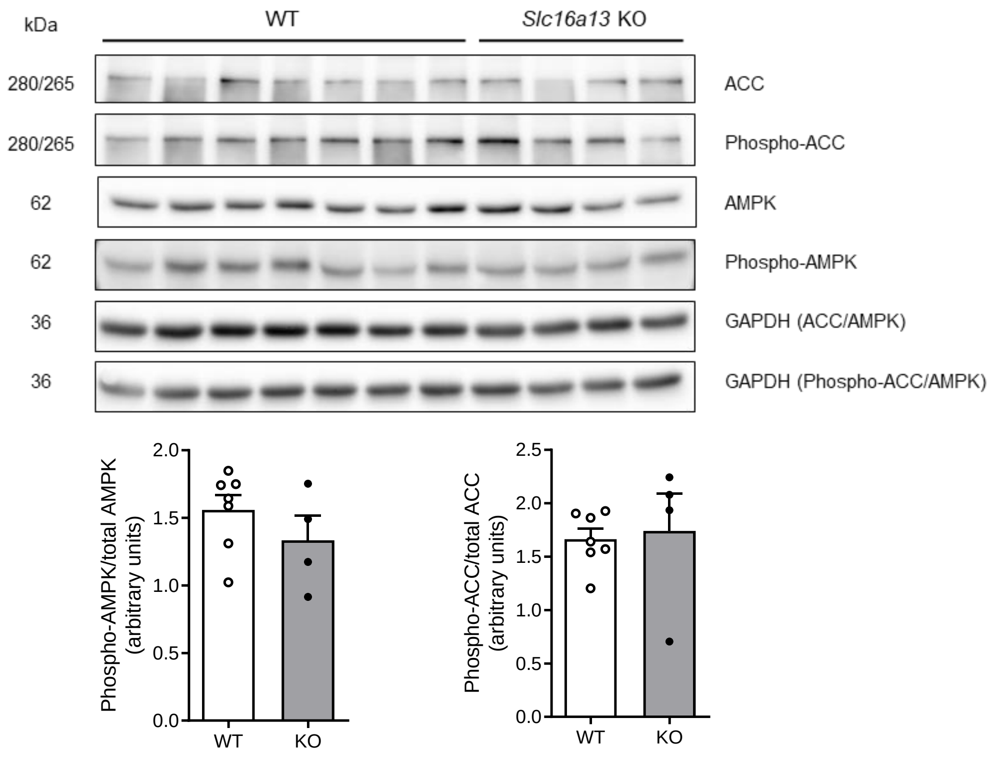

Supplementary Fig. 7 Molecular insight into increased AMPK activation in *Slc16a13* knockout primary hepatocytes. a ACC and AMPK phosphorylation of palmitate-treated hepatocytes (Western Blot and ratio of phospho-ACC/total ACC and phospho-AMPK/total AMPK determined by densitometric analysis). **b** ACC and AMPK phosphorylation of BSA-

treated hepatocytes (Western Blot and ratio of phospho-ACC/total ACC and phospho-AMPK/total AMPK determined by densitometric analysis). Hepatocytes isolated from *Slc16a13* WT (n=7) and *Slc16a13* KO (n=4) mice at a mean age of 20 weeks. Bars represent means ± SEM. *$p<0.05$ determined using two-tailed unpaired Student´s t-test.

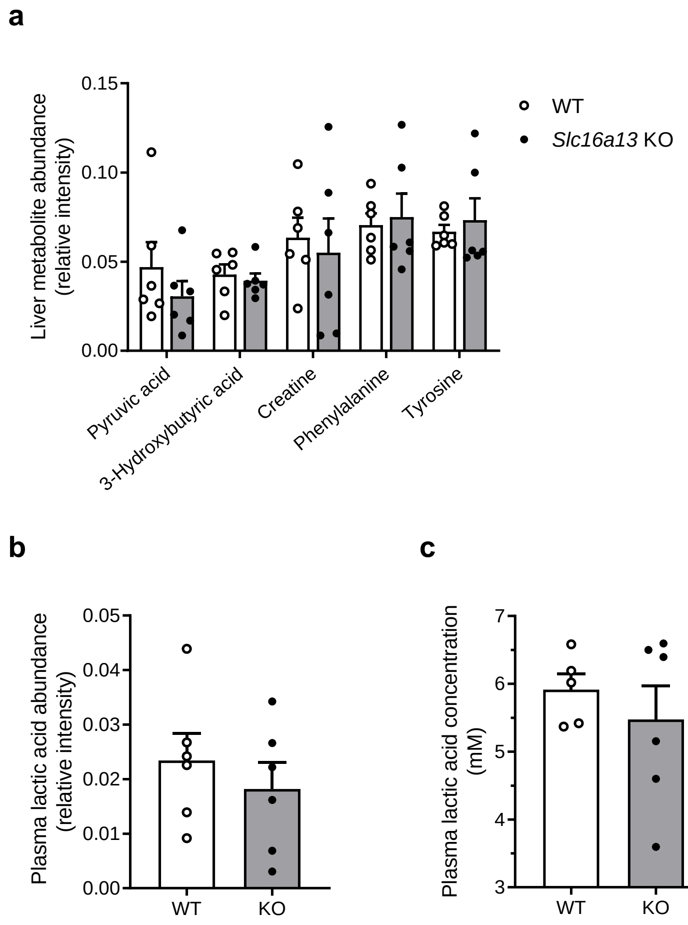

Supplementary Fig. 8 Liver and plasma metabolites of *Slc16a13* knockout mice at 15 weeks of HFD. a Relative levels of liver metabolites transported by other SLC16 family members. **b** Relative plasma lactic acid levels determined by untargeted metabolomics. **c** Plasma lactic acid concentrations determined by enzymatic assay. *Slc16a13* WT (n=5-6), *Slc16a13* KO (n=6). Bars represent means ± SEM.

a

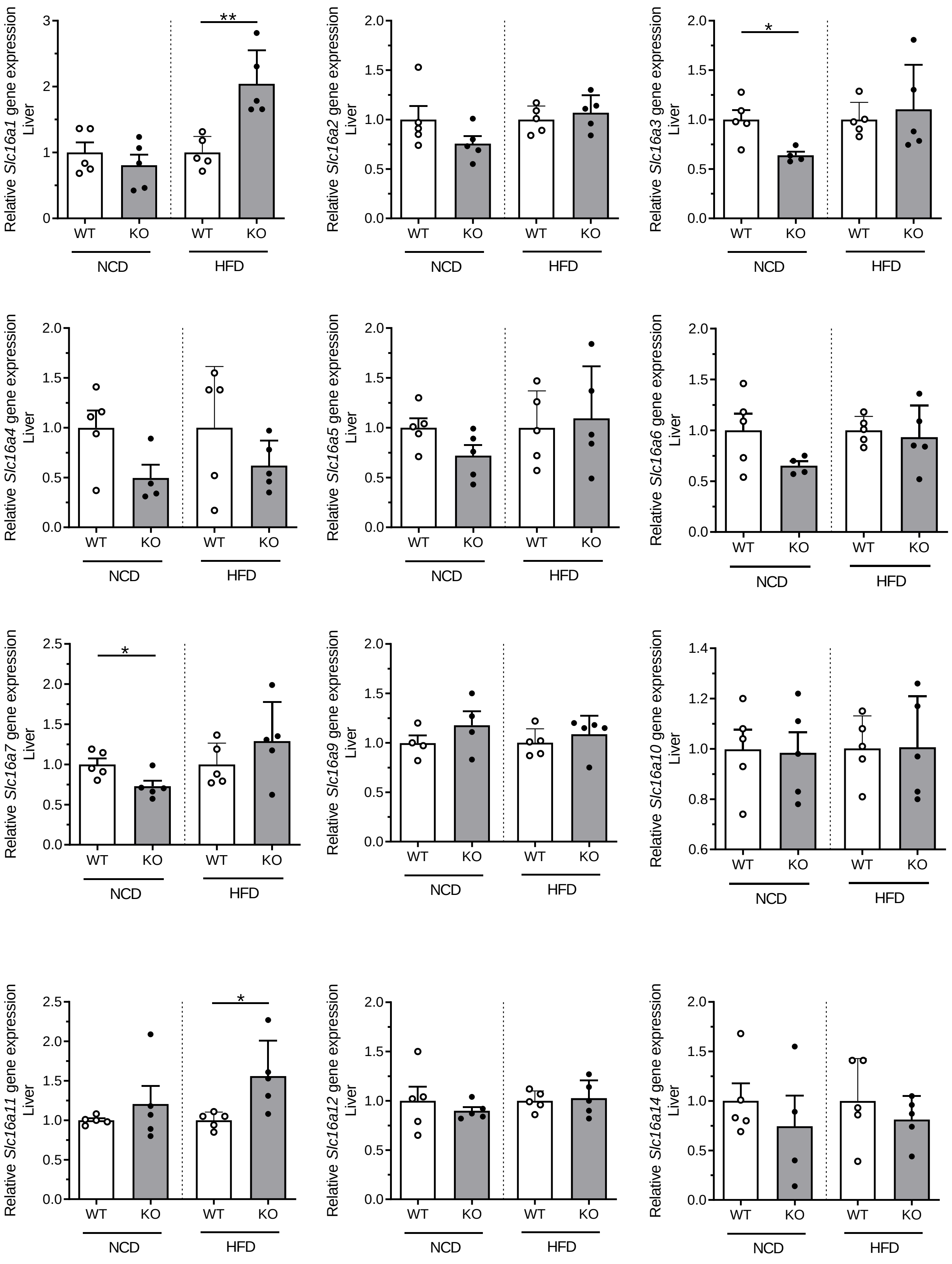
Relative *Slc16a1* gene expression Liver
Relative *Slc16a2* gene expression Liver
Relative *Slc16a3* gene expression Liver
Relative *Slc16a4* gene expression Liver
Relative *Slc16a5* gene expression Liver
Relative *Slc16a6* gene expression Liver
Relative *Slc16a7* gene expression Liver
Relative *Slc16a9* gene expression Liver
Relative *Slc16a10* gene expression Liver
Relative *Slc16a11* gene expression Liver
Relative *Slc16a12* gene expression Liver
Relative *Slc16a14* gene expression Liver
WT
KO
NCD
HFD
**
*

b

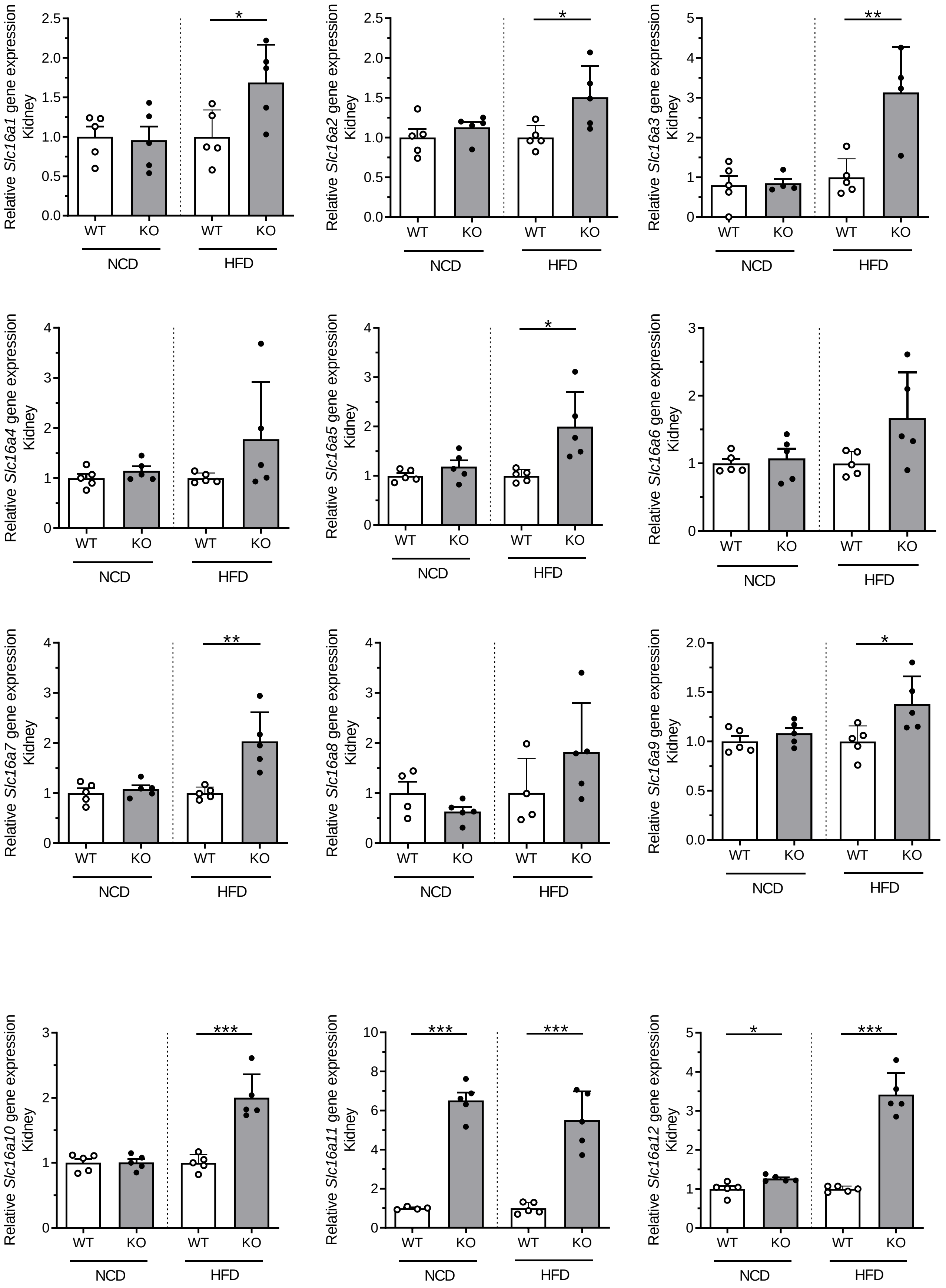
Relative Slc16a1 gene expression Kidney
Relative Slc16a2 gene expression Kidney
Relative Slc16a3 gene expression Kidney
Relative Slc16a4 gene expression Kidney
Relative Slc16a5 gene expression Kidney
Relative Slc16a6 gene expression Kidney
Relative Slc16a7 gene expression Kidney
Relative Slc16a8 gene expression Kidney
Relative Slc16a9 gene expression Kidney
Relative Slc16a10 gene expression Kidney
Relative Slc16a11 gene expression Kidney
Relative Slc16a12 gene expression Kidney
WT
KO
NCD
HFD
*
**
***

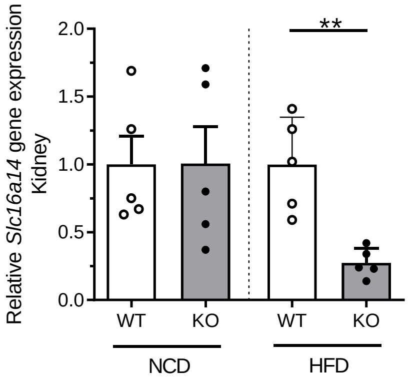

Supplementary Fig. 9 Gene expression of Slc16 family members in liver and kidney of *Slc16a13* knockout mice. Relative mRNA expression of different Slc16 genes in liver (**a**) and kidney (**b**) of NCD- and HFD-fed *Slc16a13* KO mice and WT controls (n=4-5 for each genotype). *Slc16a8* was not detectable in liver tissue. Bars represent means ± SEM. *p<0.05, **p<0.01, ***p<0.001 determined using two-tailed unpaired Student´s t-test.

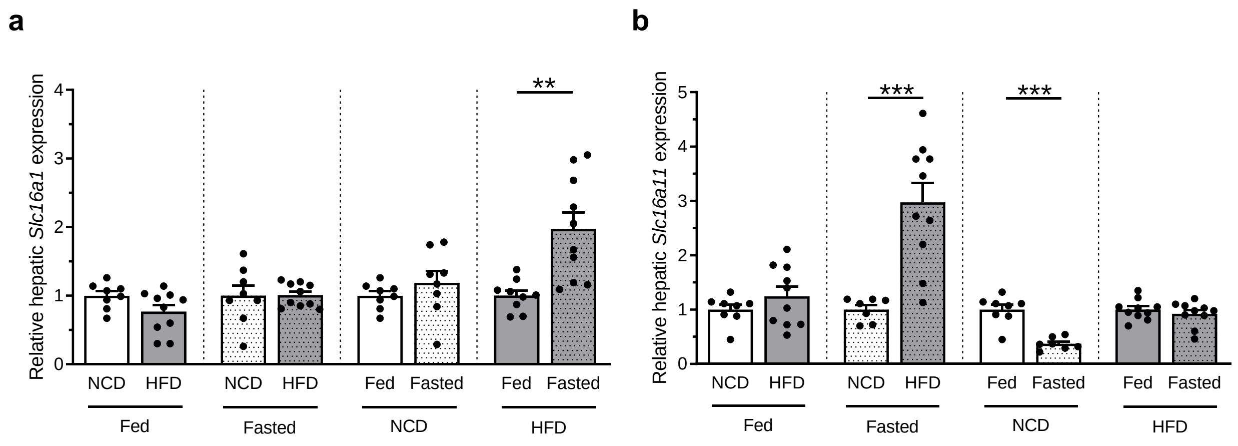

Supplementary Fig. 10 *Slc16a1* and *Slc16a11* expression in diet-induced obesity. Relative hepatic *Slc16a1* (**a**) and *Slc16a11* (**b**) mRNA expression in C57BL/6J mice fed with NCD or HFD for 15 weeks. Animals are random fed (n=7-10 per group) or fasted for 16 hours (n=6-9 per group). Bars represent means ± SEM. **p<0.01, ***p<0.001 determined using two-tailed unpaired Student´s t-test.

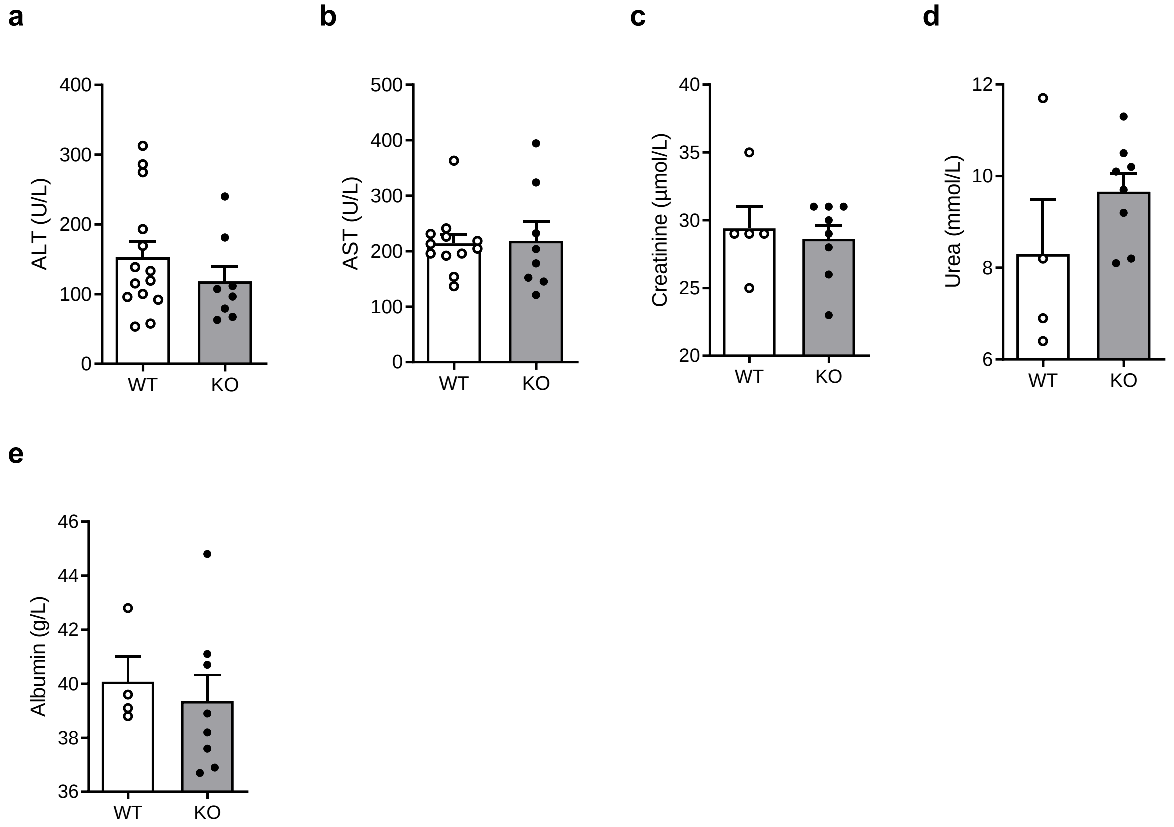

Supplementary Fig. 11 Plasma concentration of biomarkers of liver and kidney function in *Slc16a13* knockout mice at 15 weeks of HFD. a Plasma alanine aminotransferase (ALT). **b** Plasma aspartate aminotransferase (AST). **c** Creatinine. **d** Urea. **e** Albumin. *Slc16a13* WT (n=4-7), *Slc16a13* KO (n=7-8). Bars represent means ± SEM.

a

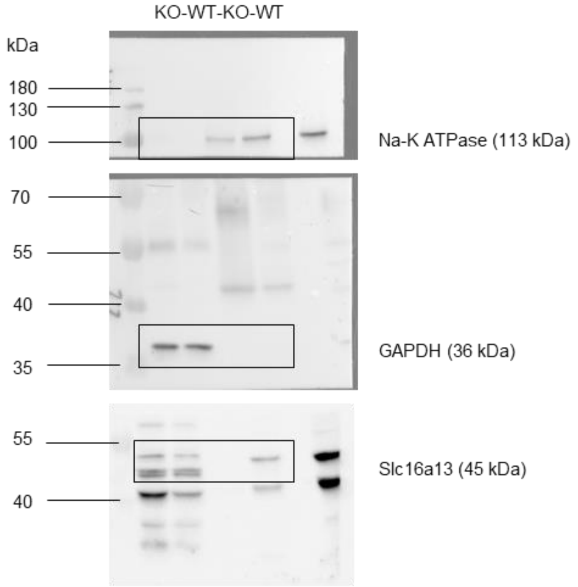

KO-WT-KO-WT
kDa
180
130
100
Na-K ATPase (113 kDa)
70
55
40
GAPDH (36 kDa)
35
55
Slc16a13 (45 kDa)
40

b

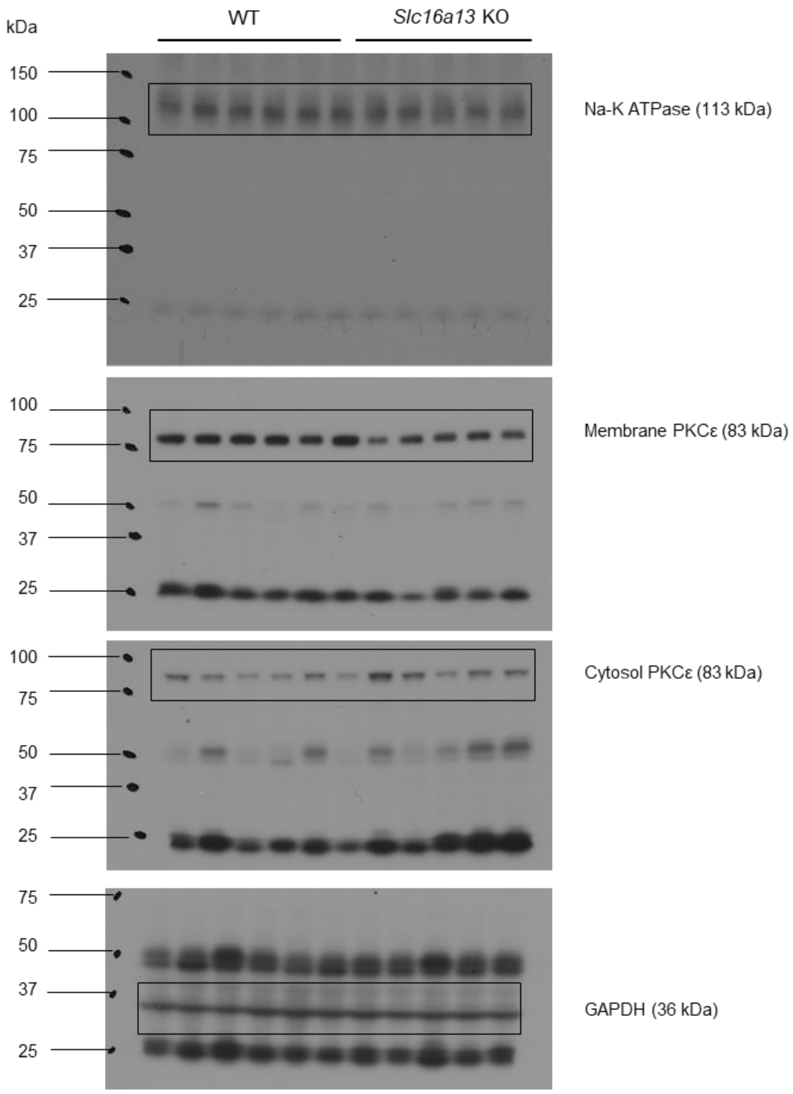

kDa
WT
Slc16a13 KO
150
100
75
50
37
25
Na-K ATPase (113 kDa)
100
75
50
37
25
Membrane PKCε (83 kDa)
100
75
50
37
25
Cytosol PKCε (83 kDa)
75
50
37
25
GAPDH (36 kDa)

c

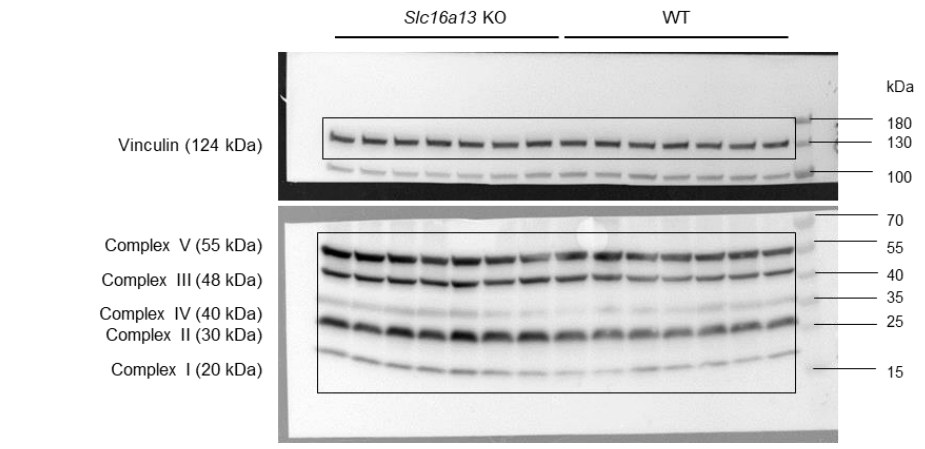

d

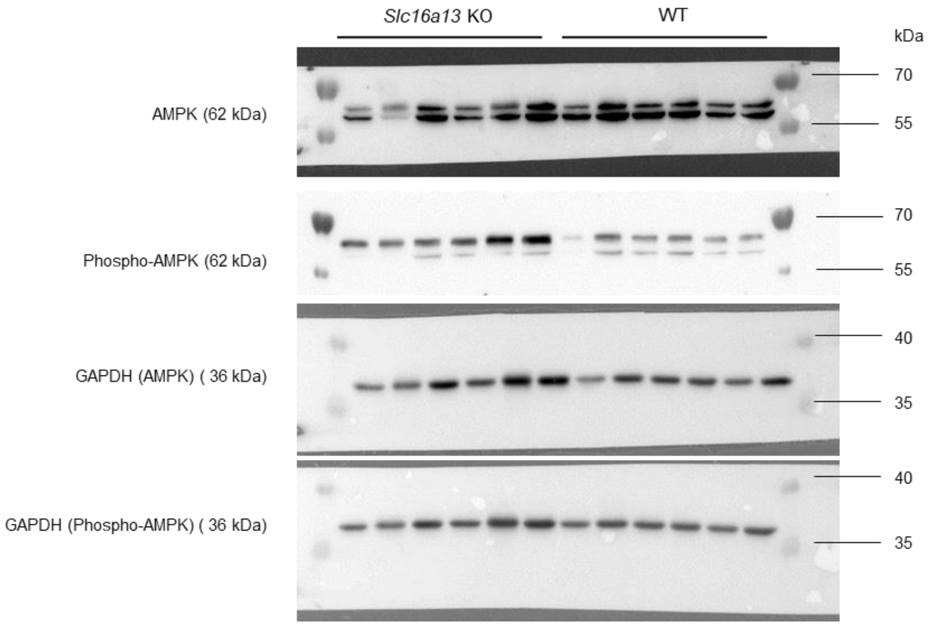

e

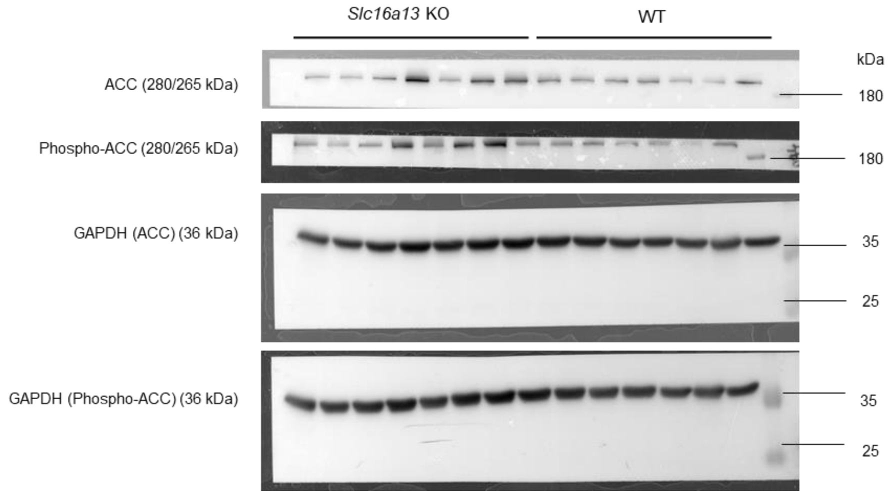

Supplementary Fig. 12 Uncropped Western blots. Uncropped merged marker images related to Fig. 3c (**a**), 7e (**b**), 9a (**c**), 9c (**d**), 9d (**e**).

Supplementary Table 1: Physical and clinical parameter of human patients. Means ± SEM are displayed. HOMA-IR, homeostasis model assessment for insulin resistance; SBP, systolic blood pressure; DBP, diastolic blood pressure.

n (male)	45 (17)
age (years)	59±2
BMI (kg/m²)	26.7±1.0
waist circumference (cm)	97±3
SBP (mm Hg)	133±3
DBP (mm Hg)	73±2
HOMA-IR	2.4±0.4
NAFLD activity score (0-8)	1.7±0.2
Liver steatosis (%)	15.9±3.2

Supplementary Table 2: Multivariate linear regression analysis of parameters related to *SLC16A13* in human liver. Significant associations are shown in bold. HOMA-IR, homeostasis model assessment for insulin resistance; TAG, triacylglycerides.

	Model 1 Dependent variable: **Waist circumference**	Model 2 Dependent variable: **BMI**	Model 3 Dependent variable: **log histological steatosis**	Model 4 Dependent variable: **log liver TAG**	Model 5 Dependent variable: **log HOMA IR**
	R = 0.622; R^2 = 0.386; adj. R^2 = 0.340; $p < 0.001$	R = 0.493; R^2 = 0.243; adj. R^2 = 0.187; $p = 0.009$	R = 0.725; R^2 = 0.525; adj. R^2 = 0.446; $p = 0.001$	R = 0.506; R^2 = 0.256; adj. R^2 = 0.166; $p = 0.039$	R = 0.499; R^2 = 0.249; adj. R^2 = 0.174; $p = 0.019$
**Independent variables:**	**β-coefficient (*p*-value)**	**β-coefficient (*p*-value)**	**β-coefficient (*p*-value)**	**β-coefficient (*p*-value)**	**β-coefficient (*p*-value)**
Age	0.100 (0.43)	-0.155 (0.27)	0.126 (0.41)	0.056 (0.73)	-0.195 (0.17)
Gender	-0.251 (**0.050**)	-0.047 (0.73)	0.009 (0.95)	0.109 (0.48)	-0.267 (0.059)
BMI	-	-	0.150 (0.37)	0.247 (0.15)	0.176 (0.27)
Liver *SLC16A13* mRNA	0.558 (**< 0.001**)	0.493 (**0.001**)	0.626 (**0.001**)	0.336 (0.065)	0.289 (0.077)

Supplementary Table 3: Body weight and relative tissue mass of NCD- and HFD-fed *Slc16a13* knockout and wild-type mice. Results are expressed as means ± SEM. *p<0.05, **p<0.01, ***p<0.001 determined using two-tailed unpaired Student´s t-test. eWAT, epigonadal white adipose tissue; reWAT, renal white adipose tissue; subWAT, subcutaneous white adipose tissue.

	normal-chow diet		high-fat diet	
	**WT (n=9)**	***Slc16a13* KO (n=11)**	**WT (n=14)**	***Slc16a13* KO (n=10)**
Body weight (g)	26.01±0.48	24.87±0.63	41.18±0.97	40.29±0.9
Liver (% of body weight)	3.89±0.06	4.04±0.13	3.18±0.14	3.22±0.21
eWAT (% of body weight)	1.77±0.20	1.11±0.13*	5.69±0.18	5.17±0.26
reWAT (% of body weight)	0.58±0.07	0.27±0.04***	2.96±0.09	2.78±0.13
subWAT (% of body weight)	0.74±0.07	0.42±0.07**	4.47±0.31	3.75±0.23
Quadriceps (% of body weight)	0.87±0.13	1.06±0.03	0.74±0.02	0.83±0.11

Supplementary Table 4: Sequences of qRT-PCR primer.

hHPRT-for	TGACACTGGCAAAACAATGCA
hHPRT-rev	GGTCCTTTTCACCAGCAAGCT
hSLC16A13-for	CAGTTTGGGAGCCCGGTAG
hSLC16A13-rev	AGGTGGGTCAAGGAAGTAGC
mCav1-for	GGGCAAATACGTAGACTCCGAGG
mCav1-rev	CTTGACCACGTCGTCGTTGAGA
mCd36-for	GATGACGTGGCAAAGAACAG
mCd36-rev	TCCTCGGGGTCCTGAGTTAT
mFabp1-for	GGAAGGACATCAAGGGGGTG
mFabp1-rev	TCACCTTCCAGCTTGACGAC
mFatp2-for	TGAATGTGTATGGCGTGCCT
mFatp2-rev	AGGTACTCCGCGATGTGTTG
mFatp5-for	TTGCATTCCTGTGGAGCCAG
mFatp5-rev	TACGCGTCGTACATTCGCAA
mHprt-for	TCCCAGCGTCGTGATTAGC
mHprt-rev	CCAGCAGGTCAGCAAAGAAC
mSlc16a1-for	AGTGCAACGACCAGTGAAGT
mSlc16a1-rev	GCGATCATTACTGGACGGCT
mSlc16a2-for	CGGCATCCATAACTCTGTTGG
mSlc16a2-rev	AGATCATGCCCATAGCGAGG
mSlc16a3-for	CTCTTTGCGTCCCTGGGAAT
mSlc16a3-rev	AAGGCTGGAAGTTGAGAGCC
mSlc16a4-for	CTCCCCTTACACCAAACCCC
mSlc16a4-rev	TGCAAATGTCTTGGTCATTCCC
mSlc16a5-for	CAGCAACAGTGAGACCTCGT
mSlc16a5-rev	GGCAGCCGAAATGTTTGACC
mSlc16a6-for	AAAGGCGTTCGGTAGTCACC
mSlc16a6-rev	GAGGCTGTATCTCCAGCCAAT
mSlc16a7-for	CACCACCTCCAGTCAGATCG
mSlc16a7-rev	CTCCCACTATCACCACAGGC
mSlc16a8-for	CTTTGGGGCAGGTTACAGTGA
mSlc16a8-rev	CAAAGCGCGTCACGAGGAT
mSlc16a9-for	AAGGAAAAACAGCTTGGGTGG
mSlc16a9-rev	GTGACGGGTCTTGCTCCAAA
mSlc16a10-for	CTGGACACCTTCAAGGCCAA
mSlc16a10-rev	GTCCGTGAAGACACTCACGAT
mSlc16a11-for	AGGCAGCCAGCCCAGT
mSlc16a11-rev	ACCAGGGCCCAGCCAGA
mSlc16a12-for	GAGGCTGGGGCTGGATGATA
mSlc16a12-rev	AGCAAAGTATGTCTGAAACTCCAC
mSlc16a13-for	AGCAGTTTGGGAGCCCAATAG
mSlc16a13-rev	AGCCAGAGCCTGACAGCA
mSlc16a14-for	CAGCCTTACTAGGACCACCA
mSlc16a14-rev	TGAGCAAAAAGAGTATCCCTACCA
