# Supplementary material for: Deletion of the diabetes candidate gene Slc16a13 in mice attenuates diet-induced ectopic lipid accumulation and insulin resistance
Source: Commun Biol. 2021 Jul 1;4:826. doi: 10.1038/s42003-021-02279-8 (PMC8249653; doi:10.1038/s42003-021-02279-8)
Supplement: Supplementary file 2 — Description of Additional Supplementary Files [file 42003_2021_2279_MOESM2_ESM.pdf]

Description of Additional Supplementary Files

File name: Supplementary Data 1

Description: All source data underlying the graphs and charts in the main figures.
