# Supplementary material for: Deletion of the diabetes candidate gene Slc16a13 in mice attenuates diet-induced ectopic lipid accumulation and insulin resistance
Source: Commun Biol. 2021 Jul 1;4:826. doi: 10.1038/s42003-021-02279-8 (PMC8249653; doi:10.1038/s42003-021-02279-8)
Supplement: Supplementary file 4 — Reporting Summary [file 42003_2021_2279_MOESM4_ESM.pdf]

Corresponding author(s): Andreas L. Birkenfeld

Last updated by author(s): Jan 19, 2021

Reporting Summary

Nature Research wishes to improve the reproducibility of the work that we publish. This form provides structure for consistency and transparency in reporting. For further information on Nature Research policies, see our Editorial Policies and the Editorial Policy Checklist.

Statistics

For all statistical analyses, confirm that the following items are present in the figure legend, table legend, main text, or Methods section.

n/a	Confirmed	
☐	☒	The exact sample size (*n*) for each experimental group/condition, given as a discrete number and unit of measurement
☐	☒	A statement on whether measurements were taken from distinct samples or whether the same sample was measured repeatedly
☐	☒	The statistical test(s) used AND whether they are one- or two-sided<br>*Only common tests should be described solely by name; describe more complex techniques in the Methods section.*
☒	☐	A description of all covariates tested
☐	☒	A description of any assumptions or corrections, such as tests of normality and adjustment for multiple comparisons
☐	☒	A full description of the statistical parameters including central tendency (e.g. means) or other basic estimates (e.g. regression coefficient) AND variation (e.g. standard deviation) or associated estimates of uncertainty (e.g. confidence intervals)
☒	☐	For null hypothesis testing, the test statistic (e.g. *F*, *t*, *r*) with confidence intervals, effect sizes, degrees of freedom and *P* value noted<br>*Give P values as exact values whenever suitable.*
☒	☐	For Bayesian analysis, information on the choice of priors and Markov chain Monte Carlo settings
☒	☐	For hierarchical and complex designs, identification of the appropriate level for tests and full reporting of outcomes
☒	☐	Estimates of effect sizes (e.g. Cohen's *d*, Pearson's *r*), indicating how they were calculated

Our web collection on statistics for biologists contains articles on many of the points above.

Software and code

Policy information about availability of computer code

Data collection	Microsoft Excel was used to collect the data.
Data analysis	Microsoft Excel and GraphPad Prism 7 were used to analyze the data. Fiji-ImageJ software (NIH) was used for densitometric analysis of Western Blots, Seahorse Wave (Agilent Technologies) and DatLab 4 software (OROBOROS Instruments) for respirometry data analysis.

For manuscripts utilizing custom algorithms or software that are central to the research but not yet described in published literature, software must be made available to editors and reviewers. We strongly encourage code deposition in a community repository (e.g. GitHub). See the Nature Research guidelines for submitting code & software for further information.

Data

Policy information about availability of data

All manuscripts must include a data availability statement. This statement should provide the following information, where applicable:

- Accession codes, unique identifiers, or web links for publicly available datasets
- A list of figures that have associated raw data
- A description of any restrictions on data availability

The data that support the findings of the study are available from the corresponding author on reasonable request.

Field-specific reporting

Life sciences study design

All studies must disclose on these points even when the disclosure is negative.

Sample size	No sample-size calculation was performed for in vitro experiments. For in vivo experiments, animal numbers were planned by a statistician using Student´s t-test or two-way ANOVA according to study design and estimated variables for each experiment. Alternatively, sample sizes were chosen based on prior research done in our laboratory.
Data exclusions	Single data points were excluded if they were significant outliers ($p<0.05$) according to Grubbs' test (GraphPad outlier calculator) as indicated in Supplementary Data.xlsx.
Replication	In vivo mouse experiments were not replicated. Different cohorts of Slc16a13 knockout and wild-type littermates were included in this study in order to conform animal welfare and secure the reproducibility of the metabolic phenotype.
Randomization	Randomization was not relevant to our study. Groups were assigned according to the genotype of the mice (WT or Slc16a13 KO).
Blinding	Human liver tissue NAFLD-activity score was determined by a sample-masked pathologist. H&E and Oil-Red-O stainings of mouse liver samples were evaluated by a sample-masked technician.

Reporting for specific materials, systems and methods

We require information from authors about some types of materials, experimental systems and methods used in many studies. Here, indicate whether each material, system or method listed is relevant to your study. If you are not sure if a list item applies to your research, read the appropriate section before selecting a response.

Materials & experimental systems

n/a	Involved in the study	
☐	☒	Antibodies
☐	☒	Eukaryotic cell lines
☒	☐	Palaeontology and archaeology
☐	☒	Animals and other organisms
☐	☒	Human research participants
☒	☐	Clinical data
☒	☐	Dual use research of concern

Methods

n/a	Involved in the study	
☒	☐	ChIP-seq
☒	☐	Flow cytometry
☒	☐	MRI-based neuroimaging

Antibodies

Antibodies used	for immunofluorescence:<br>anti-FLAG (Thermo Fisher Scientific, PA1-984B, 1:500), anti-Golgin-97 (Thermo Fisher Scientific, A-21270, 1:100), anti-Calnexin (Novus Biologicals, NB300-518, 1:100), goat anti-rabbit Alexa Fluor 488 secondary antibody (Thermo Fisher Scientific, A-11008, 1:500), goat anti-mouse Alexa Fluor 594 (Thermo Fisher Scientific, R37121, 1:500), Alexa Fluor 594 wheat germ agglutinin (Thermo Fisher Scientific, W11262, 1:100)<br>for Western Blot:<br>anti-SLC16A13 (Thermo Fisher Scientific, PA5-39249, 1:500), anti-Vinculin (Abcam, ab129002, 1:10000), anti-GAPDH (Abcam, ab8245, 1:20000), anti-Na-K ATPase (Abcam, ab7671; 1:1000), anti-PKCε (BD Biosciences, 610086, 1:1000), anti-Acetyl Coenzyme A Carboxylase (Abcam, ab45174, 1:2000), anti-Acetyl Coenzyme A Carboxylase (phospho S79) (Abcam, ab68191, 1:5000), total OXPHOS Rodent WB Antibody Cocktail (Abcam, ab110413, 1:1000), anti-AMPK alpha 1 + AMPK alpha 2 antibody (Abcam, ab207442, 1:1000), anti-AMPK alpha 1 (phospho T183) + AMPK alpha 2 (phospho T172) (Abcam, ab133448, 1:1000), anti-Akt (pan) (Cell Signaling, 4691, 1:1000), anti-Akt (phospho Ser473) (Cell Signaling, 4060, 1:1000), goat anti-rabbit IgG, HRP conjugated (Millipore, 401353, 1:10000), goat anti-mouse IgG, HRP conjugated (Millipore, 401253, 1:10000)
Validation	Antibodies were validated for their application by the manufacturer.

Eukaryotic cell lines

Policy information about cell lines

Cell line source(s)	HEK293 cells were obtained from ATCC.
Authentication	Cell lines have been authenticated by ATCC and in our laboratory based on cellular morphology.
Mycoplasma contamination	Cell lines were not tested for mycoplasma contamination.
Commonly misidentified lines<br>(See ICLAC register)	No commonly misidentified cell lines were used in this study.

Animals and other organisms

Policy information about studies involving animals; ARRIVE guidelines recommended for reporting animal research

Laboratory animals	Male WT and Slc16a13 KO mice on C57BL/6N background (Taconic Biosciences) were used for all studies, except for data based on mouse primary hepatocytes that were isolated from female mice. The specific diet started at 5 weeks of age, experiments were performed at 12-20 weeks of age.
Wild animals	The study did not involve wild animals.
Field-collected samples	The study did not involve samples collected from the field.
Ethics oversight	All procedures were approved by the Landesdirektion Sachsen in accordance with national guidelines.

Note that full information on the approval of the study protocol must also be provided in the manuscript.

Human research participants

Policy information about studies involving human research participants

Population characteristics	Characteristics of the 45 included patients from the cross-sectional INSIGHT-study are shown in Supplementary Table 1.
Recruitment	Liver biopsies were taken from patients undergoing partial hepatectomy. All subjects gave written informed consent at least 24 hours prior to surgery.
Ethics oversight	Informed consent was obtained from each patient included in the study, and the study protocol conforms to the ethical guidelines of the 1975 Declaration of Helsinki as reflected in approval by the ethics committee of the Charité-Universitätsmedizin Berlin.

Note that full information on the approval of the study protocol must also be provided in the manuscript.
